# Developmentally relevant proteins contribute to chemotherapeutic resistance in neuroblastoma

**DOI:** 10.3389/fonc.2026.1926773

**Published:** 2026-09-24

**Authors:** Nina F. Schor

**Affiliations:** Departments of Pediatrics and Neurology, University of Rochester School of Medicine & Dentistry, Rochester, NY, United States

**Keywords:** histone deacetylase inhibitor, neural crest development, neural differentiation, neuroblastoma, neurotrophin receptors, protein methylation, transcription factors

## Abstract

**Background:**

Facilitators and suppressors of oncogenesis during childhood include many of the proteins and processes critical to development. Neuroblastoma is a childhood cancer that arises from the cells in the neural crest destined to populate the sympathetic nervous system. Among the arbiters and effectors of development in the neural crest are the neurotrophin receptors, Trks A, B, and C and p75NTR; the transcription factors, SIX1 and ATF5; the tyrosine kinase, ALK; and the histone modifiers, EZH2 and the HDACs. During neural development, the relative cellular expression, activation, and translocation of these proteins play important roles in determination of cell fate. We hypothesize that these proteins also play important roles in the chemoresistance of neuroblastoma.

**Objective:**

This review explores existing evidence to implicate these developmentally relevant proteins and their temporal and spatial inconstancy in the challenge of chemoresistance of neuroblastoma.

**Literature review methods:**

For examination of the role of developmentally relevant mechanisms in neuroblastoma resistance to chemotherapy, searches of PubMed and ClinicalTrials.gov databases were performed using the following search terms: “neurotrophin receptor” AND “neuroblastoma”; “Trk” AND “neuroblastoma”; “p75NTR” AND “neuroblastoma”; “EZH2 inhibitors” AND “neuroblastoma”; “HDAC inhibitors” AND “neuroblastoma”; “epigenetic” AND “neuroblastoma” AND “resistance”; “ALK inhibitors” AND “neuroblastoma”; “PRMT” AND “neuroblastoma”; and “neuroblastoma differentiation” AND “therapy”. Papers published within the past 10 years were considered for inclusion in discussion of response of neuroblastoma cells and clinical tumors to chemotherapeutic agents and clinical studies closed to recruitment within the past 5 years or continuing to recruit were considered for inclusion in discussion of future therapeutic approaches to clinical neuroblastoma.

**Literature evidence synthesis:**

A total of 317 papers were identified across all initial topics and, of these, 195 were found to be relevant to neuroblastoma therapy. Eleven papers were identified as relevant to 2 or more topics (8 papers, 2 topics; 2 papers, 3 topics; 1 paper, 6 topics), resulting in 175 unique papers identified *in toto*. The literature supports the notion that developmentally driven heterogeneity and changeability of specific protein expression, activation, and translocation in neuroblastomas contributes to the refractoriness of neuroblastoma to chemotherapy.

**Conclusions:**

Therapeutic approaches aimed at inhibition or epigenetic modification of intratumoral neurotrophin receptors, transcription factors, tyrosine kinases, and histone modifiers are currently in clinical trials for neuroblastoma. Future efforts will require both individualization of therapy and emerging technological approaches to the heterogeneity and inconstancy of expression and processing of developmentally relevant proteins in neuroblastoma.

## 1 Introduction

Cancer in adults is envisioned as the result of environmental “hits” to fully differentiated, mature cells. The term “dedifferentiation” has been used in some circumstances (1); the notion of a predisposing genetic or other endogenous characteristic with a “second hit” inducing cancer in susceptible individuals has been implicated in others (2). Cancer in young children, and particularly in neonates and infants, is more often envisioned as the result of developmental arrest of undifferentiated cells, often associated with genetic or epigenetic abnormalities of regulators of differentiation and protein expression within a particular lineage (3). As such, variability in the stage of differentiation at which the cells undergo oncogenic developmental arrest and retain the potential to continue to proliferate and metastasize, adopting in new environments developmentally different transcriptomes, presents unique challenges for identifying therapeutic targets for effective chemotherapy of childhood cancer. This review illustrates these challenges and some of the attempts to overcome them using neuroblastoma, a common cancer that arises from the cells of the developing neural crest, as an example of a developmental cancer of childhood.

## 2 Literature review methods

For examination of the role of developmentally relevant mechanisms for neuroblastoma resistance to chemotherapy, searches of PubMed and ClinicalTrials.gov databases were performed using the following search terms: “neurotrophin receptor” AND “neuroblastoma”; “Trk” AND “neuroblastoma”; “p75NTR” AND “neuroblastoma”; “EZH2 inhibitors” AND “neuroblastoma”; “HDAC inhibitors” AND “neuroblastoma”; “epigenetic” AND “neuroblastoma” AND “resistance”; “ALK inhibitors” AND “neuroblastoma”; “PRMT” AND “neuroblastoma”; and “neuroblastoma differentiation” AND “therapy” (**Figure 1**). Papers published within the past 10 years were considered for inclusion in discussion of response of neuroblastoma cells and clinical tumors to chemotherapeutic agents and clinical studies closed to recruitment within the past 5 years or continuing to recruit were considered for inclusion in discussion of future therapeutic approaches to clinical neuroblastoma. A total of 317 papers were identified across all initial topics and, of these, 195 were found to be relevant to neuroblastoma therapy. Eleven papers were identified as relevant to 2 or more topics (8 papers, 2 topics; 2 papers, 3 topics; 1 paper, 6 topics), resulting in 175 unique papers identified *in toto* (**Figure 2**; **Table 1**). All 175 papers were reviewed in drawing the conclusions of this review. However, single case reports and overarching review articles were only cited and included in the References section if they provided information or ideas not reflected in the primary research, well-powered studies identified.

**Figure 1:**
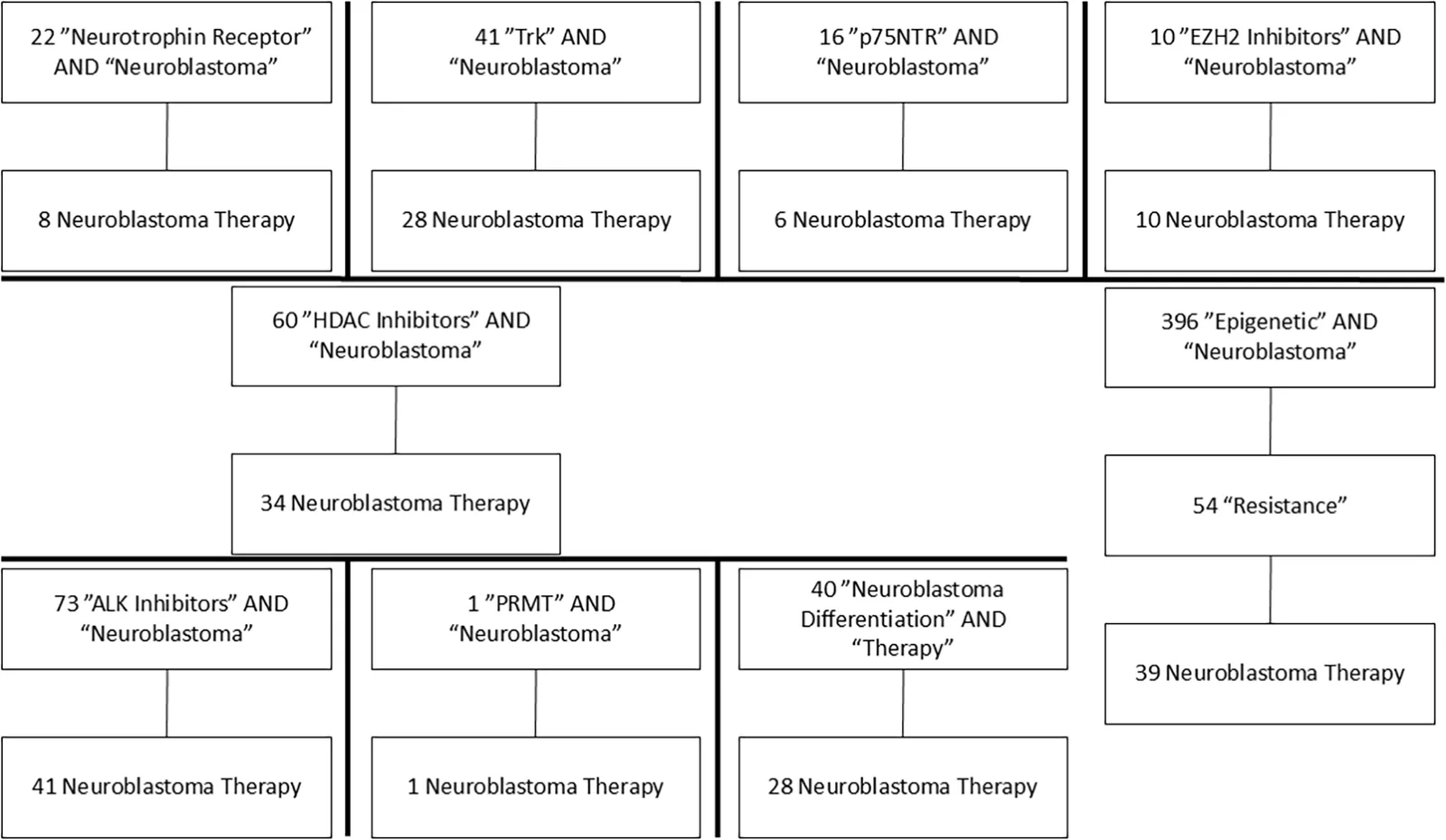
Literature search results for each search category. In each vertical pair of boxes, the number of articles identified in the initial search category and the number of those articles related to neuroblastoma therapy, respectively, are shown.

**Figure 2:**
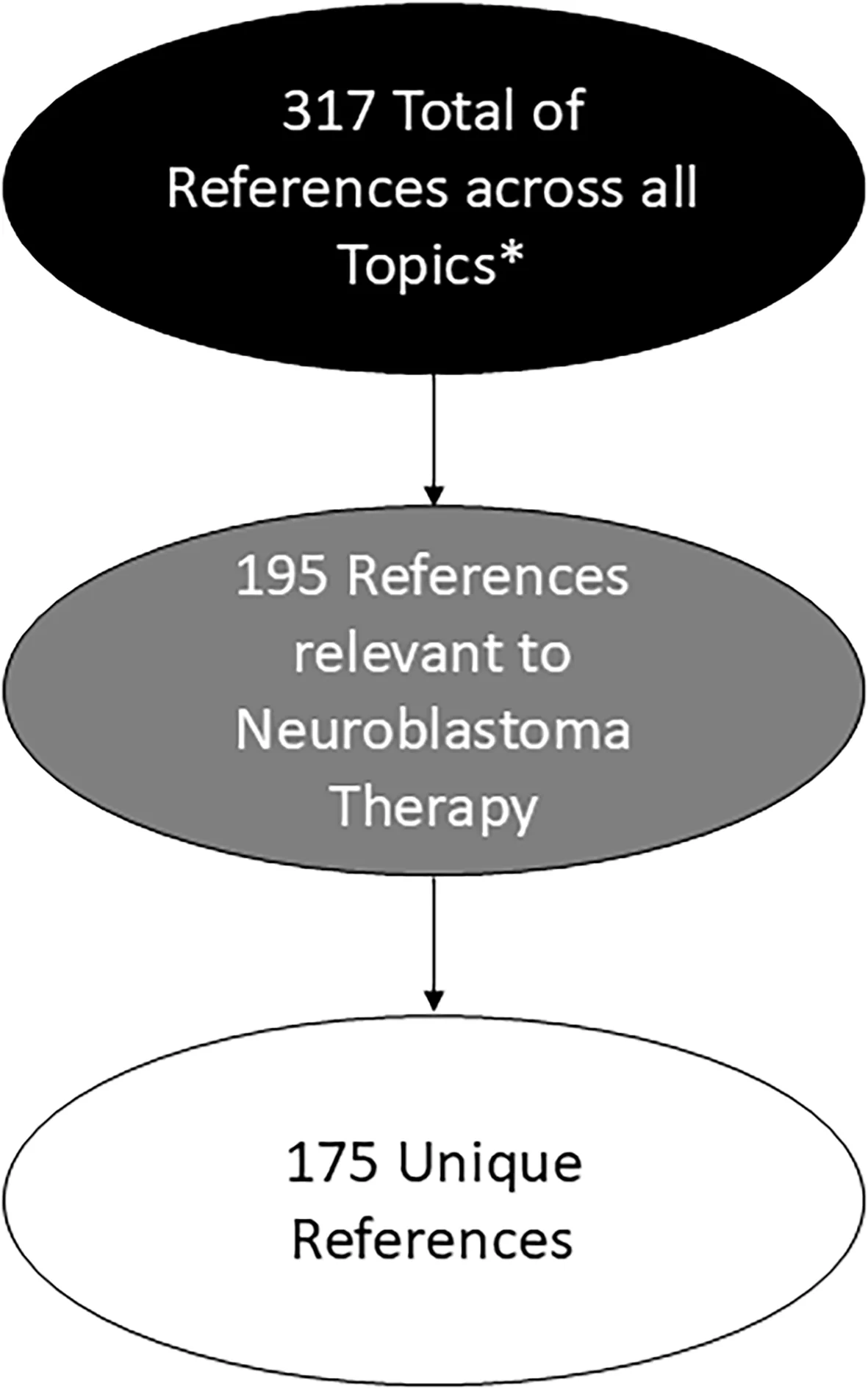
Aggregate literature search results for all categories. Shown are the arithmetic sum of all articles identified in all categories shown in **Figure 1** without accounting for the appearance of some articles in multiple categories; the arithmetic sum of the subset of all articles identified in all categories shown in **Figure 1** that are related to neuroblastoma therapy; and the number of all articles identified as being related to neuroblastoma therapy that does not count any article more than once (i.e., unique articles). *Of the 396 articles identified in “Epigenetic” AND “Neuroblastoma”, only the 54 of them identified in a subsearch for “Resistance” are included.

**Table 1:** Pairs of categories in which one or more identified references are shared (i.e., duplicative references).

Search Categories	ALK	EZH2	HDAC	Differentiation	Epigenetic resistance	Neural crest development	Neurotrophin receptors	p75 NTR	PRMT	Trk
ALK		0	0	0	1	0	0	0	0	0
EZH2	0		0	0	1	0	0	0	0	0
HDAC	0	0		0	2	0	2	3	0	1
Differentiation	0	0	0		2	0	0	0	0	0
Epigenetic Resistance	1	1	2	2		0	0	1	0	1
Neural Crest Development	0	0	0	0	0		0	0	0	0
Neurotrophin Receptors	0	0	2	0	0	0		3	0	2
p75NTR	0	0	3	0	1	0	3		0	1
PRMT	0	0	0	0	0	0	0	0		0
Trk	0	0	1	0	1	0	2	1	0	

Background information on developmentally relevant mechanisms was obtained from references identified by the following searches in the PubMed database: “ALK” AND “neural crest development”; “EZH2” AND “neural crest development”; “HDAC” AND “neural crest development”; “p75NTR” AND “neural crest development”; “SIX1” AND “neural crest development”; and “Trk” AND “neural crest development”. In addition, some papers referenced in the works identified through PubMed are also cited.

## 3 Neuroblastoma: a developmental cancer of childhood

### 3.1 Embryological and clinical aspects

Neuroblastoma is the single most common solid tumor of childhood. It arises from the neuroblastic precursor cells in the neural crest that ultimately migrate out to form the sympathetic nervous system, including the neuroendocrine component of the adrenal gland. As such, neuroblastoma can occur anywhere along the sympathetic nervous system axis. Approximately 50% of primary tumors arise in the adrenal gland and 25% occur elsewhere in the abdomen. The remaining primary tumors are found in the chest and neck. The median age at diagnosis is 17 months (4).

Unlike adult cancers, thought to arise from fully differentiated cells that undergo malignant transformation which restores or augments proliferative potential, neuroblastoma can be conceived to be an anomaly of cellular maturation with its cells “stuck” in an intermediate stage of differentiation (3). As neural crest cells migrate out to form the sympathetic ganglia at each level along the body’s rostro-caudal axis, they progress through a differentiation program, including an epithelial-to-mesenchymal transition, modulated by the environment through which they are migrating. Each level’s environment and each stage in the process of migration induces differential expression of cellular proteins and a different morphological and functional array of characteristics (5). This heterogeneity of expression, morphology, and function over time and space and the plasticity of both the cells and their environment contribute to the challenge of designing targeted therapy for neuroblastoma.

Extent and severity of disease in patients with neuroblastoma have been characterized in the International Neuroblastoma Risk Group Staging System (**Table 2**). This rubric is used to gauge how aggressive the upfront therapeutic approach to a given patient’s disease needs to be to optimize the prognosis for that patient. Other factors, including the patient’s age at diagnosis; the tumor histology, degree of differentiation, and ploidy; amplification status of the oncogene *MYCN*; and presence of 11q chromosomal aberrations, are used to augment the accuracy of prediction of the aggressiveness of disease and the consequent need for aggressive initial therapy.

**Table 2:** International neuroblastoma risk group staging system [constructed from (4)].

Designation	Description
Stage L1	Localized tumor; no vital structures involved; tumor confined to 1 major body compartment; no image-defined risk factors (N.B.: Intraspinal extension that does not meet criteria for image-defined risk factors is consistent with Stage L1 designation.)
Stage L2	Locoregional tumor; evidence of image-defined risk factors; contiguous disease across more than 1 major body compartment is solely unilateral (N.B.: Non-contiguous disease should be designated as Stage M.)
Stage M	Distant metastatic disease in patient who does not meet criteria for Stage MS; non-contiguous disease; distant lymph node involvement
Stage MS	Patients < 18 months (547 days) old with metastatic disease limited to: • Bone marrow with negative MIBG scintigraphy of bone and bone marrow • Skin • Liver

Body compartments include neck, chest, abdomen, pelvis; MIBG, radiolabeled metaiodobenzylguanidine used to identify neuroendocrine tumors; image-defined risk factors include encasement of major vessels or invasion of vital structures.

Prognosis for children with neuroblastoma is roughly bimodal in several respects. Children under the age of 1 year at diagnosis have better survival rates than older children at any stage of disease. This is best illustrated by comparing children with Stage M disease metastatic to the bone marrow, skin, or liver (50% 5-year overall survival) with children with Stage MS disease (98% 5-year overall survival). In children with Stage MS disease, total resection of the primary tumor is most often followed by regression of the metastases. Children whose tumors overexpress the oncogene *MYCN* have a much worse 5-year survival rate than those who do not overexpress *MYCN*. Children with low or intermediate risk (Stages L1 or L2) have a 90-95% 5-year overall survival rate while children whose tumors have metastasized to sites remote from the primary tumor (Stage M) have an approximately 50% 5-year overall survival rate (4).

### 3.2 Challenges of therapeutic resistance of neuroblastoma

There are many characteristics of neural crest development and differentiation that contribute to the chemoresistance of the neuroblastoma cells derived from the neural crest. First, as neural crest cells migrate from the edge of the neural plate to their ultimate destinations, they undergo epithelial-to-mesenchymal transition. This process involves an epigenetic sequence of changes in expression of a host of transcription factors that results first in delineation, respectively, of the neurons and glia of the sensory ganglia; the autonomic-mesenchymal progenitor cells; the osteoclasts and chondrocytes; and the melanocytes. A second transformation involves a split of the autonomic-mesenchymal progenitor cells into the mesenchymal and sympatho-adrenal lineages, also facilitated by epigenetic changes in transcription factor expression. The sympatho-adrenal progenitor cells thus derived are the cells of origin of neuroblastomas. Depending on the nature and stage of differentiation of these cells of origin, both intra- and extra-adrenal neuroblastomas can arise from sympathetic neural-type, glial-type, or sympathoblastic cells or, in the adrenal medulla, chromoffin neuroendocrine cells, each with a unique expression profile and, therefore, a unique set of chemotherapeutic targets and susceptibilities. Both chemotherapeutic selection for one lineage over another and the epigenetic potential of these cell types to transform from one to another also play roles in chemoresistance of neuroblastoma (5, 6).

The fact that many of the proteins involved in the process of differentiation and oncogenic transformation of the sympatho-adrenal lineage are transcription factors makes them challenging targets, as well, as each of these transcription factors has many and varied downstream targets that might influence differently the outcome of altered transcription factor expression. In addition, the oncogene *MYCN* is expressed in primitive neural crest cells and downregulated as the epithelial-to-mesenchymal transition progresses but then is re-expressed in sympatho-adrenal progenitor cells. This re-expression is thought to contribute to both oncogenesis and subsequent virulence and chemoresistance in this lineage (6).

In addition to the expression changes that occur during neural crest cell migration and differentiation, sympatho-adrenal lineage cells express neurotrophic receptors and downstream signal transductants that mediate resistance to oxidative stress and life-or-death decision-making in the cell. To the extent that many of these are initiators or effectors of developmentally important protective and/or proliferative pathways, they play a role, as well, in resistance to cytotoxic chemotherapeutic agents (7).

In the subsequent sections of this review, several of the developmentally relevant proteins that mediate chemotherapeutic resistance of neuroblastoma are discussed. Many of these are putative targets for adjunctive therapy; others have mechanisms that are either just beginning to be elucidated or exhibit sufficient plasticity of expression or epigenetic modification to make them challenging as therapeutic targets. In the respective discussions of receptors and enzymes, transcription factors, and histone modifiers that follow, the roles of these proteins in normal neural crest development are presented and the current state of understanding of their role in chemoresistance of neuroblastoma is discussed. Finally, for each, the existing, proposed, and envisioned approaches to overcoming their respective contributions to chemoresistance are presented.

## 4 Developmentally relevant mediators of therapeutic resistance in neuroblastoma: neurotrophin receptors

### 4.1 Developmental expression and function of neurotrophins and their receptors

Differentiation and sustenance of the cells of the neural crest is largely mediated by two neurotrophin receptors and their respective cognate ligands. TrkA is a high-affinity receptor, encoded by the *NTRK1* gene, that binds nerve growth factor (NGF). TrkA-NGF is the classical mediator of neuroblast differentiation and survival. Signaling through the NGF-TrkA-Ras-Raf-MEK1-ERK1/2-CREB pathway mediates this nurturant effect of the TrkA-NGF pair (7).

The p75 neurotrophin receptor (p75NTR; encoded by the *NGFR* gene) has a more complex functional story. Originally conceived as only a “co-receptor” that, when bound to TrkA, enhanced the affinity of TrkA for NGF, p75NTR was subsequently found to also function as an independently signaling receptor. This latter function is predominantly as a “death receptor” and p75NTR has been shown to bind to the precursor protein of NGF, proNGF, in the presence of the protein sortilin to induce apoptosis in neural crest-derived cells through a signaling pathway involving NF-κB and c-IAP2. Consequently, p75NTR, through its cooperation with TrkA, can serve to enhance the nurturant effects of NGF or, through its independent signaling, can induce the death of neural elements. Which function p75NTR serves is determined by the relative abundance of p75NTR and TrkA in the cell membrane and the concentration of NGF and its precursor, proNGF in the cellular environment (7, 8).

Both TrkA and p75NTR play critical roles in the proliferation, neural differentiation, and sustenance of neural crest cells. In addition, other neurotrophin receptors play important roles in nervous system development and maintenance. TrkB is encoded by the *NTRK2* gene and binds brain-derived neurotrophic factor (BDNF). Similarly to TrkA, TrkB partners with p75NTR increasing its affinity for BDNF. TrkC is encoded by the *NTRK3* gene. The cognate ligand for TrkC is neurotrophin-3 (NT-3). TrkB-BDNF and TrkC-NT-3 initiate signaling in the developing neural crest that is temporally restricted by the respective windows of expression of TrkB and TrkC. Furthermore, p75NTR can independently bind to the neurotrophins BDNF, NT-3, and NT-4/5, earning it designation as a “promiscuous” neurotrophin receptor (7).

TrkA can also bind NT-3, albeit with low affinity. During sympathetic nervous system development, increased expression of TrkA induces activation of Arf6, a GTPase, that, in turn, induces trafficking of p75NTR from intracellular locations to the cell membrane, where it binds to TrkA. Unlike TrkA alone, the TrkA-p75NTR complex does not bind NT-3 but binds NGF with highest affinity. This developmental sequence shifts neurotrophic signaling in sympathetic neuroblasts from NT-3 to NGF (9).

Just as is the case for p75NTR, which can play pro- or antiapoptotic roles depending on its expression ratio relative to other neurotrophin receptors in a particular cell (8), overexpression of TrkA shifts downstream signaling of the TrkA-NGF pair from the antiapoptotic NGF-TrkA-MEK1-ERK1/2-CREB pathway to the proapoptotic NGF-TrkA-MEK3/6-p38 MAP pathway (10).

#### 4.1.1 TrkA, TrkB, and TrkC developmental expression and signaling

*NTRK1*, the gene that encodes TrkA, is expressed in cranial and spinal sensory ganglia and paraspinal sympathetic neurons throughout life (11, 12). The TrkA-NGF complex signals primarily through 3 pathways (**Figure 3**). The Ras and PI3K pathways primarily promote cell survival while the phospholipase C-γ (PLCγ) pathway primarily promotes neuronal differentiation and neurite outgrowth. Downstream of the protein scaffold-mediated activation of Ras, the Raf protein kinase activates the MEK family kinase cascade which ultimately results in activation of transcription factors (MYC, Elk1, and CREB) that upregulate expression of pro-survival proteins. Through the phosphoinositol-3-kinase (PI3K) pathway, Akt activation leads to initiation of the MAPK pathway through the GTP binding protein Rac and also results in activation of nuclear transcription factors that upregulate expression of pro-survival proteins. Secondarily, Akt activation also leads to induction of the PKC pathway. The PKC pathway fosters neuronal differentiation and neurite outgrowth and is primarily initiated by activation of PLCγ (12).

**Figure 3:**
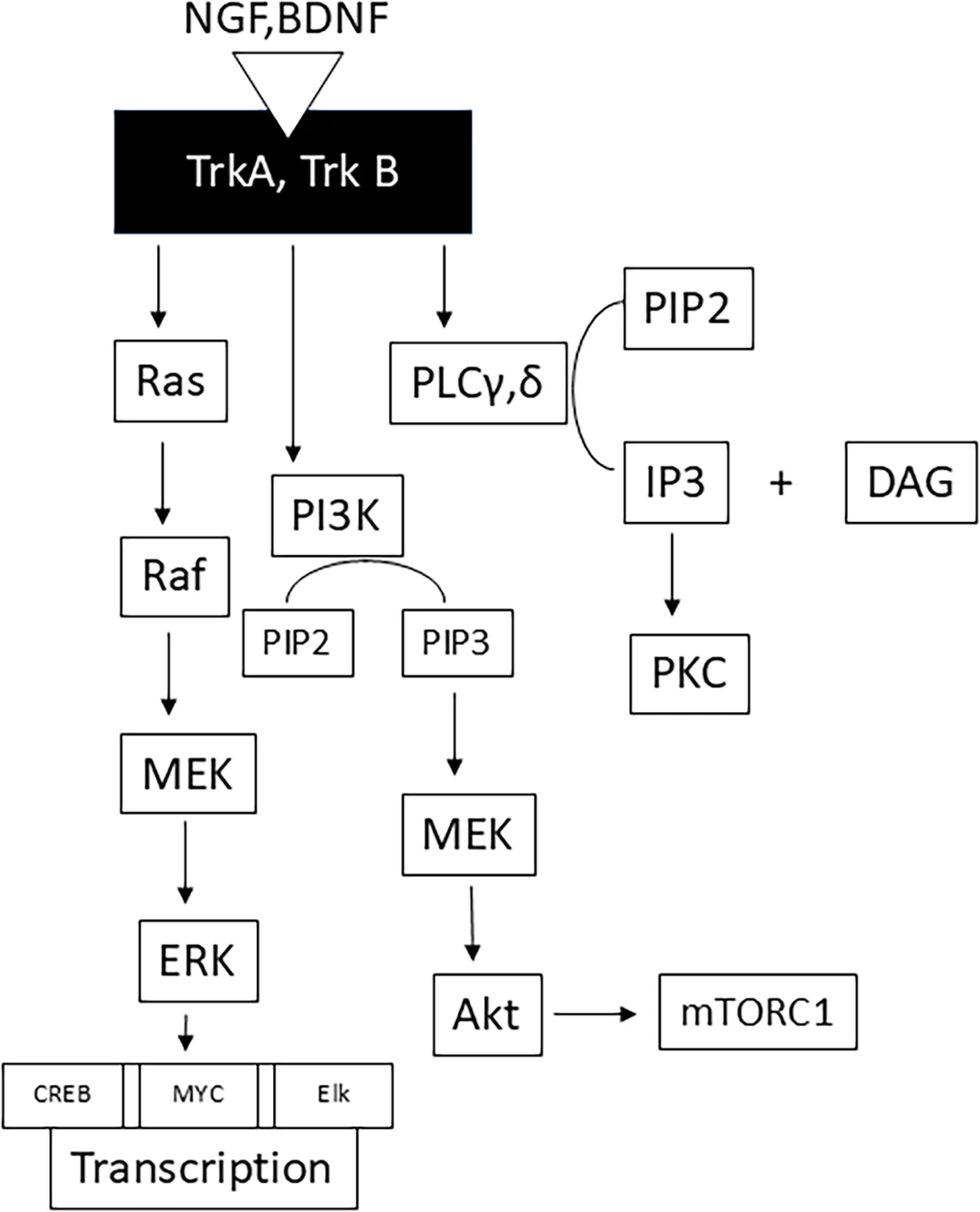
Three signaling pathways most commonly initiated by NGF or BDNF binding to TrkA or TrkB, respectively. Standard signal effector abbreviations are defined in the text.

Most TrkB-expressing cells in the early-stage embryo are of placode, not neural crest, origin. However, there is a temporal window, during differentiation of neural crest cells into sympathetic neurons and subsequent ventral migration of clusters of these cells to form the sympathetic ganglia, when cells of neural crest origin do express TrkB (13–15). From studies originally done in the chick and more recently done in zebrafish, TrkB-BDNF signaling enables cell migration and appears to have been leveraged and sustained beyond its normal temporal window in metastatic neural crest-derived cancers, including melanoma and neuroblastoma (14). In the central nervous system, TrkB-BDNF signaling proceeds through the Ras-ERK, PI3K, and phospholipase C-δ (PLCδ) pathways (**Figure 3**) and, as such, also supports neuronal survival and process outgrowth (16).

Developing sympathoblasts appear to include early-appearing cells that express both TrkA and TrkC but not TrkB and later-appearing cells that do express TrkB. In culture, the former population of cells is dependent on NGF and NT-3 for proliferation and survival. Like other Trk receptors, TrkC signaling involves the PI3K/AKT, MAPK/ERK, and PLCγ pathways (15, 16).

#### 4.1.2 p75NTR developmental expression and signaling

As a binding partner with many other proteins, p75NTR is perhaps the most complex and multifaceted of the neurotrophin receptors (**Figure 4**). It functions as a coreceptor for their respective cognate ligands with each of the Trk receptors. To a first approximation, the binding of p75NTR to any of the Trk receptors enhances the Trk receptor affinity for its ligand and thereby renders signaling through the Trk receptor more potent in its nurturant and differentiative functions (17).

**Figure 4:**
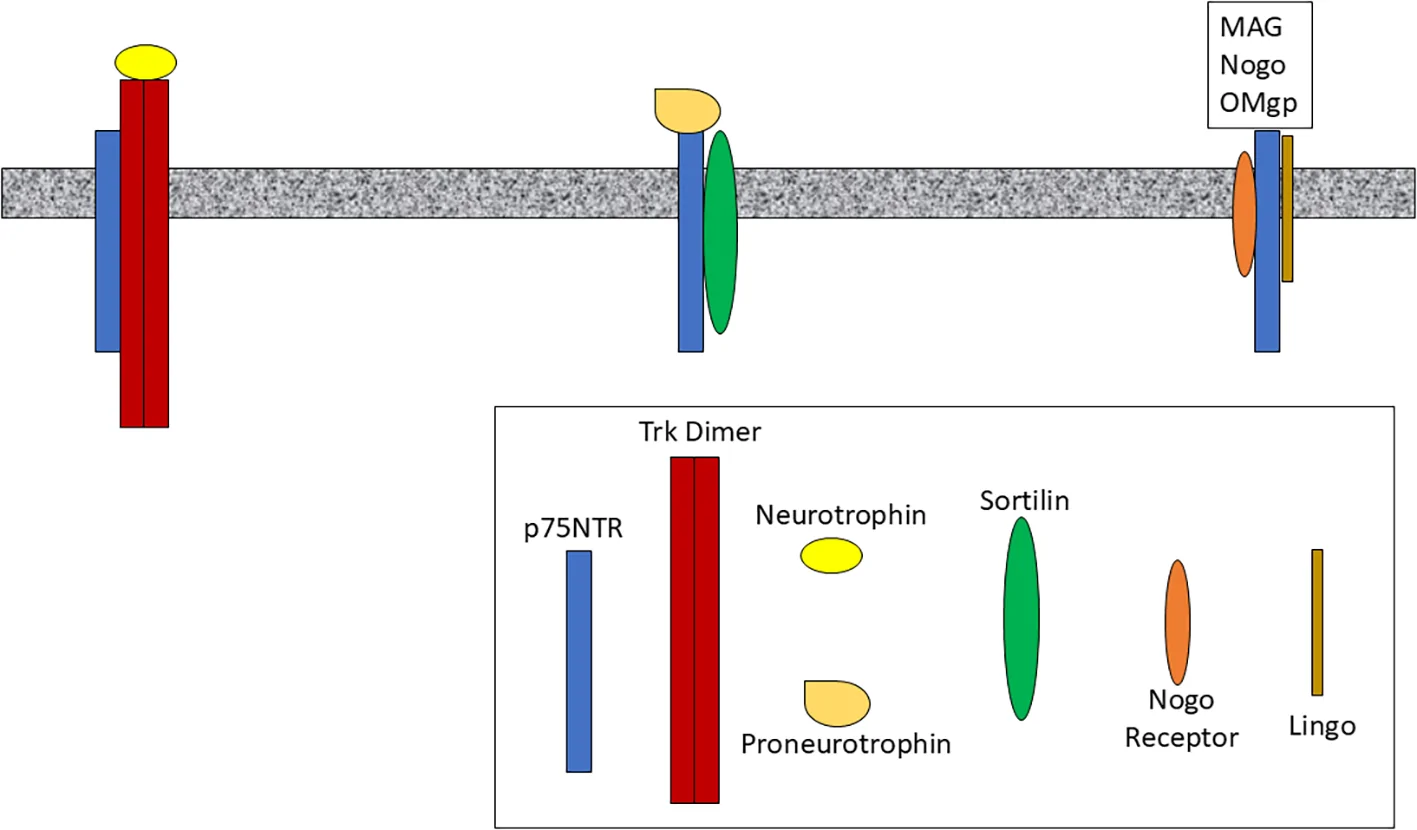
Binding partners and ligands of the p75NTR. MAG, myelin associated glycoprotein; OMgp, oligodendrocyte myelin glycoprotein.

In addition, p75NTR partners with sortilin to bind proNGF or proBDNF. In contrast to binding of NGF or BDNF to a Trk-p75NTR complex, this coupling leads to apoptosis. The p75NTR also partners with the Nogo family receptors to bind myelin-associated glycoprotein, oligodendrocyte myelin glycoprotein, and the transmembrane protein Nogo-A, inhibiting axon outgrowth (18). Both sortilin and Nogo coupling of p75NTR are thought to affect neural crest cell migration, sortilin enhancing it and Nogo inhibiting it (17).

Each of the varied effects of p75NTR and one of its diverse partners is dependent on complex formation with one or more of a large array of other proteins. For example, coupling of the p75NTR-TrkB-BDNF complex with the protein Kidins220/ARMS that has been phosphorylated by the protein Ephrin B is thought to facilitate the cytoskeletal remodeling that accompanies migration and neurite outgrowth during epithelial-to-mesenchymal transition (17, 19, 20).

### 4.2 Putative roles of neurotrophins and their receptors in chemoresistance of neuroblastoma

At the simplest level, empiric observation of patients with neuroblastoma has led to the general conclusions that high levels of TrkA or TrkC in a tumor give the patient a more favorable prognosis, while high levels of TrkB confer a poorer prognosis. TrkA- and TrkC-high tumors are more likely to undergo neural differentiation or to spontaneously regress, while TrkB-high tumors are more likely to undergo transition to a mesothelial (stromal) lineage and to therefore be more resistant to chemotherapy, more likely to induce vascularization, and more likely to metastasize. Expression of p75NTR in tumors with high TrkA or TrkC expression enhances the likelihood of tumor differentiation or regression (7).

Attempts to leverage these observations to therapeutic advantage have met with several challenging characteristics of the developmental-oncological transformation of neural crest cells. First, each of the Trk receptors and the p75NTR receptor has an effect on cell survival, mitotic potential, and differentiation that is dependent on the environment – both membrane and extracellular – in which it sits (10, 21). Second, the oncogenic transformation from neural crest cell to neuroblastoma cell is associated with epigenomic phenomena including methylation of histones, non-coding regulatory DNA sequences, overexpression and post-translational modification of the transcription factors N-myc and SIX1, and modulation of chromatin structure by small, non-coding RNAs (7, 22, 23). As a consequence, attempted targeting of mutant DNA sequences or proteins is often thwarted by their variable structural modification. Third, there is ample evidence that the cellular stress of chemotherapy can induce alternate splicing of TrkA, resulting in ligand-independent, constitutive signaling through the PI3K-Akt, anti-apoptotic, angiogenic, and metastatic pathway (24, 25). Finally, the stage of maturation of neuroblastoma cells can differ from cell to cell in the same tumor and from site to site in the case of metastases of the primary tumor (26), making it challenging to ablate a tumor by targeting a tractably-sized set of cellular proteins.

#### 4.2.1 Intra- and extracellular context-dependence of Trk and p75NTR signaling and neuroblastoma chemoresistance

Trk and p75NTR expression of neuroblastoma influences such things as tumor cell proliferation and apoptosis, inflammatory and immunologic response to the tumor, migration of tumor cells, and angiogenesis within the tumor. Not only is expression of neurotrophin receptors and neurotrophins variable from cell to cell within a single tumor; in addition, the signaling pathway initiated by a given receptor is also variable depending on structural and quantitative expression characteristics of the receptor, which are in turn influenced by the primary or metastatic environment and prior chemotherapeutic agent exposure (7, 21, 24, 25).

In primary neuroblastoma tumors, high expression of TrkB enhances angiogenesis and decreases responsiveness to chemotherapeutic agents. Cells are induced to progress along a mesenchymal-like, stromal lineage and respond to binding of the mature (i.e., post-transcriptionally processed) BDNF to TrkB with ERK, AKT, and mTORC1 pathway activation. Stabilization of Bcl-2, MCL-1, and BCL-xL protects against apoptosis. Proliferation, chemoresistance, and vascularization result (7, 19, 20).

On the other hand, high expression of TrkA enhances differentiation and neurite outgrowth and decreases proliferation, lowering the replication index of the tumor and rendering it “more benign”, with a better prognosis. As discussed in section 4.1, overexpression of TrkA can shift the preponderant downstream signaling pathway and induce cellular apoptosis and consequent tumor regression, rather than differentiation (10).

Nowhere is the relationship between the peritumoral environment and tumor responsiveness to chemotherapy more apparent than in the case of Stage MS neuroblastoma. When neuroblastoma occurs in children under the age of 18 months and involves a small primary tumor with metastases solely to liver, bone marrow, and/or skin (i.e., Stage MS neuroblastoma), the tumor burden is highly likely to regress spontaneously with little or only severe symptom-directed chemotherapy. These tumors are likely to have high levels of TrkA. On the other hand, when neuroblastoma occurs in children of the same age but involves a small primary tumor with metastases to organs other than liver, bone marrow, and/or skin (i.e., Stage M neuroblastoma), multimodal therapy is warranted and, even with it, the prognosis is poor. These tumors are likely to have high levels of TrkB (27, 28).

As further demonstration of the importance of host characteristics and tissue environment in neuroblastoma cell fate and patient prognosis determination, one study compared the expression profiles of Stage MS and Stage M neuroblastomas from patients under the age of 18 months (29). The highest differences in expression included genes that play a role in various mechanisms of cell death, with Stage MS tumors having a profile associated with susceptibility to apoptosis and ferroptosis. In addition, genes that encode for proteins in the extracellular space and matrix and lumen of the endoplasmic reticulum play major roles in defining the differences between Stage MS and Stage M neuroblastomas.

Despite the important role of extracellular (i.e., tissue environmental) proteins in differentiating Stages MS and M, it is not certain whether the expression profile determines in what tissues metastases implant or, conversely, the environment of the tissues that support implantation evokes a unique expression profile. It is clear that there is a robust interaction between neuroblastoma cells and the normal cells that surround them and secrete proteins into the environment they share. The nature of this interaction and these environmental cues, along with the endogenous expression by specific neuroblastoma cells of a particular complement of cell surface receptors, determine the effectors and downstream effects of neurotrophic signaling on the survival, proliferation, and migration of the cancer cells. The variability of signaling events and cellular outcome dependent on the complex algorithm of environmental and intratumoral circumstances makes it challenging to arrive at a single enhancing or suppressive regimen for therapy of neurotrophin-reponsive tumors. In Section 4.2.3, another mechanism is discussed by which the intra- and extracellular circumstances modify signal transduction in neuroblastoma and thereby alter tumor chemosensitivity and consequent patient prognosis.

#### 4.2.2 Epigenetic modification of Trk expression

In neuroblastomas, the preponderant mechanisms for modulation of relative neurotrophic receptor expression involve epigenetic changes to somatic cells. The *NTRK* genes are particularly susceptible to methylative repression at cytosines in their CpG-rich regions. In addition, the repressive histones that bind to all the *NTRK* genes and the *NGFR* gene are characterized by protein methylation by PRMTs. Furthermore, the N-myc protein, the cellular level of which is the most potent determinant of tumor malignancy and patient prognosis in neuroblastoma, is a transcriptional repressor of the *NTRK1* and *NGFR* genes. N-myc thereby prevents neuroblastoma cell differentiation and apoptosis, contributing to sustenance of the malignant phenotype (7).

PRMTs also activate the phosphatase EYA1 by arginine methylation (**Figure 5**). Activated EYA1 then dephosphorylates the transcription factor SIX1, and the Me-EYA1-SIX1 complex is critically important for cranial neuroblast differentiation and cranial neural crest maturation into sensory organs during development. However, the *MYCN* oncogene, the gene product of which is the N-myc oncoprotein, is also transcriptionally promoted by the Me-EYA1-SIX1 complex. PRMTs also methylate N-myc protein, a process that greatly prolongs the half-life of N-myc in the cell (22, 23, 30). Accumulation of N-myc results, intensifying repression of *NTRK1* and *NGFR*. (See Section 5.).

**Figure 5:**
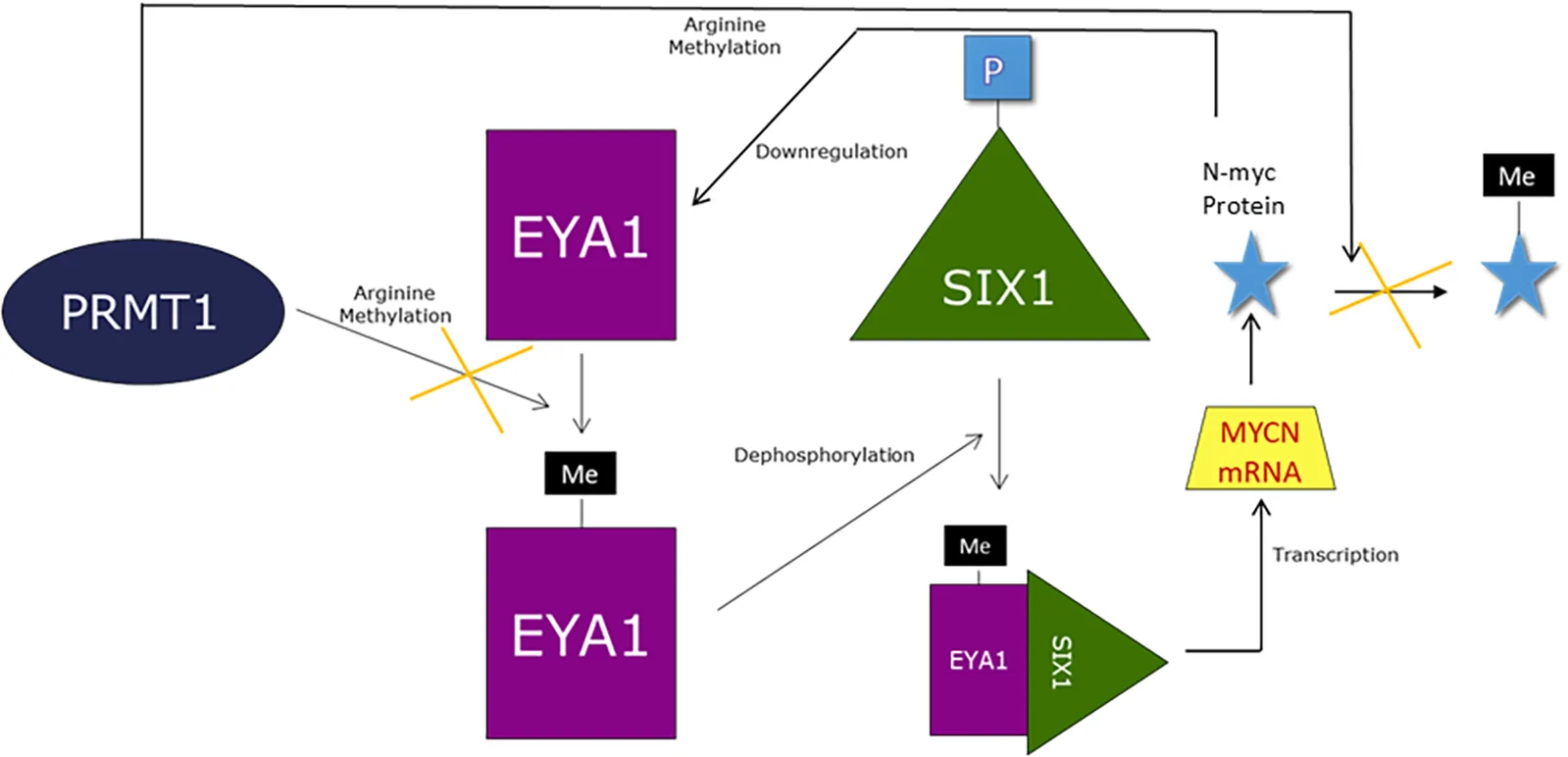
The PRMT1-EYA1-SIX1 pathway. Red Xs represent points at which PRMT1 inhibition blocks this pathway.

#### 4.2.3 Induction by cellular stress of alternative splicing of TrkA

Many factors, including tumoral hypoxia, inflammation, and oxidative effects of chemotherapy, invoke a stress reaction in neuroblastoma cells. Under circumstances of cellular stress, and particularly in recurrent neuroblastoma in patients who have undergone chemotherapeutic treatment for their initial presentation with neuroblastoma, an alternative splice variant of TrkA, called TrkAIII, can be produced. TrkAIII is missing exons 6, 7, and 9 (25).

Unlike native TrkA, which binds NGF and thereby initiates signal transduction that induces differentiation and confers good prognosis upon the patient, TrkAIII initiates ligand-independent, constitutive signaling that prevents apoptosis and sustains proliferation and confers poor prognosis upon the patient. While native TrkA is inserted into the cell membrane and exhibits extra- and intracellular domains, TrkAIII is missing the D4 immunoglobulin-like extracellular domain and binds to pre-Golgi and mitochondrial membranes, but not the cell membrane. TrkAIII also lacks several TrkA sites of N-glycosylation and is misfolded, triggering an unfolded protein response in the cells. In neuroblastoma cells that express TrkAIII, Bcl-2 is upregulated and constitutive signaling occurs through PI3K and Akt, rendering the cells resistant to chemotherapy (24). This becomes somewhat of a self-perpetuating process, as oxidative therapeutic agents, including doxorubicin, select for and reinforce the production of TrkAIII in these recurrent and often metastatic neuroblastomas (31).

#### 4.2.4 Heterogeneity of cellular stage of maturation and consequent Trk and p75NTR expression and effects of signaling

The same plasticity that makes neural crest cells and their lineage descendants so diverse in form and function and so adaptable in circumstances requiring repair, migration into and differentiation appropriate to different environments, and bidirectional transformation analogous to epithelial-mesenchymal transition makes neuroblastoma cells a challenging therapeutic target. First, as neural crest cells migrate peripherally to form the sympathetic nervous system, their transcriptome and epigenetic complement of modifications continue to change (32, 33). The differentiation state of neuroblastoma cells can vary along this entire spectrum, depending on the differentiation stage at which maturation arrest and oncogenic transformation has occurred. In addition, within a single tumor, neighborhoods of transcriptionally and epigenetically different clones occur as a result of both microenvironmental modulation, particularly in the case of metastatic disease, and chemotherapeutic selection for and resultant expansion of pre-existing molecular phenotypes. Both the expression of Trks and p75NTR and their predominant downstream effectors vary within a single patient’s tumor burden as a consequence of this clonal plasticity and heterogeneity (7).

### 4.3 Overcoming the challenges of neurotrophin-related chemotherapeutic resistance in neuroblastoma

#### 4.3.1 Induction of neural differentiation and enhancement of TrkA and TrkC expression

From the prognostic standpoint, neuroblastomas that contain more neural lineage-differentiated cells are less likely to have retained proliferative potential and more likely to express the favorable neurotrophin receptor biomarkers TrkA and TrkC, conferring a better prognosis upon the patient host (34). As a result, several therapeutic strategies for neuroblastoma have focused on induction of differentiation.

The best studied of these involves induction of neural differentiation using agonist ligands of the retinoic acid receptors (35). Both all-trans retinoic acid (ATRA) and 13-cis retinoic acid (isotretinoin) have been extensively studied *in vitro* and in subsequent clinical trials of patients with neuroblastoma. Isotretinoin has been preferred for clinical use because it is better tolerated in children. Efficacy has been variable from study to study, likely as a result of varied doses and dose schedules, formulations, and formulation- and patient-related absorption, distribution to tumor, and systemic metabolism to active metabolites. Nonetheless, the preponderance of the evidence has indicated that event-free survival and response rates for patients treated with isotretinoin maintenance therapy after chemotherapeutic ablation and stem cell transplantation are improved relative to those with chemotherapeutic ablation and stem cell transplantation alone. Isotretinoin maintenance therapy is standard for children with advanced stage neuroblastoma who have responded well to chemotherapeutic ablation followed by stem cell transplantation.

The challenges of attaining and sustaining optimal pharmacokinetics and pharmacodynamics of systemic isotretinoin concentrations have led to ongoing efforts to identify novel retinoids and formulations, including incorporation in capsules, nanoparticles, or liposomes, that improve upon the long-term steady state concentrations attainable with isotretinoin (35).

#### 4.3.2 Induction of apoptosis through p75NTR signaling

In the course of studies of alternatives to isotretinoin, another retinoid, fenretinide, was found not to induce differentiation, but rather, to induce apoptosis. Studies of the mechanism by which fenretinide exerts its cytotoxic effects demonstrated that, unlike the case of differentiation induction by isotretinoin, induction of apoptosis by fenretinide is not dependent on cellular retinoic acid binding protein 1 (CRABP1) but is dependent on expression of p75NTR and intracellular generation of reactive oxygen species. From a signaling standpoint, p75NTR-induced enhancement of apoptosis induction by fenretinide is dependent on phosphorylation of p38MAPK and JNK, activation of caspase 3, and cleavage of Akt. In addition, treatment of neuroblastoma cells with fenretinide results in upregulation of MEK4 and MEK kinase-1 (MEKK1) and phosphorylation of MEK3/6. These signaling events further distinguish isotretinoin mechanistically from fenretinide. As is the case for differentiation-inducing retinoids, novel formulations of fenretinide, including immunotargeted nanoparticle and liposomal formulations, are currently undergoing development and preclinical testing (36, 37).

## 5 Developmentally relevant mediators of therapeutic resistance in neuroblastoma: SIX1 and ATF5 transcription factors

### 5.1 Developmental expression and function of SIX1 and ATF5

Although the transcription factor SIX1 is widely expressed in the early embryo, it is best studied and functions as embryo development proceeds in its role in development of the bony structures of the face that develop from the first 2 branchial arches and the otic placodes (38, 39). In *Drosophila*, SIX1 is needed for eye development as well, but in vertebrates, it is neither involved nor necessary for such. It is also expressed and involved in development of the kidney (40). SIX1 induces transcription of proteins responsible for regulation of early osteogenesis and chondrogenesis of the mandible and middle ear ossicles. The EYA1-SIX1 complex acts as a transcription factor that, among other things, serves to repress WNT signaling by upregulation of the WNT repressor, *Frzb* (38).

Mutations of SIX1 in humans cause the range of facial and renal abnormalities that comprise the branchio-oto-renal syndrome (38–40).

The activated transcription factor ATF5 also plays an important role in embryonic development and is involved in regulation of differentiation of cells in the brain, bone, liver, and fat. It is a member of the bZip transcription factor family, as it contains a leucine zipper through which it forms homo- and heterodimers and a basic N-terminal region that binds to DNA. In the mouse brain, knockout of ATF5 results in greatly decreased proliferation of subventricular neural progenitor cells and, while ATF5 is highly expressed in periventricular neural progenitor cells in the rat, differentiation and neurite outgrowth of these cells is accompanied by downregulation of ATF5. In PC12 cells, a line derived from the neural crest adrenal tumor, pheochromocytoma, ATF5 suppresses neurite outgrowth. Furthermore, it has been shown to suppress neural, oligodendroglial, and astrocytic differentiation in normal neural progenitor cells, as well (41).

In sum, ATF5 is a pro-proliferative, pro-survival protein in neural progenitor cells (41), serving to suppress differentiation and the post-mitotic state of cells in the nervous system.

### 5.2 Putative role of SIX1 and ATF5 in chemoresistance of neuroblastoma

SIX1 expression has been demonstrated in many malignant tumor types, including neuroblastoma. In this regard, SIX1 and its various binding partners and co-transcription factors are key players in maintenance of stemness, epithelial-to-mesenchymal transition, and tumor resistance to radiation and chemotherapy (23, 30, 42–45).

In neuroblastomas and in the developing *Drosophila* eye, as is described in Section 4.2.2. and depicted in **Figure 5**, EYA1, a phosphatase that is activated by methylation by the enzyme PRMT1, dephosphorylates and then binds to SIX1. *MYCN*, the gene that encodes N-myc, in turn, a chemoresistance and aggressiveness factor in neuroblastoma, is a transcriptional target of the SIX1-EYA complex. Accordingly, tumors that overexpress *MYCN* also overexpress *PRMT1* and *Eya1* (23, 30). In addition, as discussed in Section 4.2.2., methylation of N-myc by PRMT1 prolongs the half-life of N-myc and leads to its accumulation in neuroblastoma cells, whether or not they overexpress *MYCN* at baseline (22, 23, 30).

ATF5 is also expressed in a wide variety of cancer cell types. It has perhaps been best studied in this regard in glioma cells. Gliomas that are high ATF5 expressors are among the most anaplastic and chemoresistant gliomas and auger among the worst prognoses for their patient hosts (41). In neuroblastomas, downregulation of ATF5 produces cellular apoptosis and ATF5 is highly expressed in *MYCN*-overexpressing tumors (46). Interestingly, Hua et al. (47) observed that ATF5 and PRMT1 are co-regulated and that PRMT1 knockout or inhibition results in cell death in both *MYCN*-overexpressing and -non-overexpressing neuroblastomas. This effect of PRMT1 appears not to be the result of methylation of ATF5, but rather, of modulation by PRMT1 of H4R3me2a mark at the *ATF5* gene and consequent activation of *ATF5* transcription.

### 5.3 Overcoming the challenges of SIX1- and ATF-5 -related chemoresistance in neuroblastoma

Given the epigenetic and post-translational contributors to the oncogenic signature of neuroblastoma, it is not surprising that there have been attempts to inhibit the growth of and cure patients with neuroblastoma using inhibitors of the developmentally important process of protein methylation. PRMTs are grouped by the stereochemistry and number of the methyl groups they add to proteins. Type 1 PRMTs add one or two asymmetrically placed methyl groups to arginine residues. Preclinical studies of Type 1 PRMT inhibitors and neuroblastoma have resulted in a robust understanding of their structure-anti-neuroblastoma activity relationship in cell line neurospheres and in a murine model in which, as is the case in some human neuroblastomas, *MYCN* drives malignant transformation of tyrosine hydroxylase-positive cells. It is interesting that, rather than differentiation, inhibition of PRMTs in these models results in apoptosis of neuroblastoma cells. This perhaps suggests that, although PRMT1 is responsible for increased production and prolongation of the half-life of N-myc, it is the effect of inhibition of PRMT1 on production of ATF5 that is the overriding mechanism of tumor suppression by inhibitors of PRMT1 (23, 47).

At least one proprietary inhibitor of PRMT1 (a Type 1 PRMT) has been studied in a Phase 1 clinical trial of adults with various types of solid tumors. While PRMTs are important in both central and peripheral nervous system development, and PRMT1-mediated methylation of EYA1 and N-myc, along with histone modification at *ATF5*, play roles in neuroblastoma virulence and chemotherapeutic resistance, this study included no patients with neuroblastoma or other childhood tumors, only 2 patients (6% of total sample) with a neural crest-derived tumor type (melanoma), and no patients with tumor types shown to have been driven by N-myc or ATF5. Nonetheless, it is instructive that dose-limiting toxicity in the form of thromboembolic events prevented progression to examination of the efficacy of this agent and that solid tumor delivery and penetration seemed to be limited (48).

## 6 Developmentally relevant mediators of therapeutic resistance in neuroblastoma: ALK tyrosine receptor kinase

### 6.1 Developmental role of ALK

Receptor tyrosine kinases (including the Trk receptors already discussed) play critical roles in development. These enzymes include the subset of anaplastic lymphoma kinases (ALKs) and the related leukocyte tyrosine kinases (LTKs). They have been studied in zebrafish, frogs, chickens, mice, and humans and show remarkable evolutionary conservation (49–51). In mammals, *LTK* is expressed in pre-B cells and B cells and in brain, while *ALK* is expressed in the central and neural crest-derived peripheral nervous system, including the sympathetic ganglia.

Functionally, *ALK* serves during development to enable migration of neural crest cells that have detached from the extracellular matrix and to prevent apoptosis of these cells during migration through the mesothelium (50). A family of small protein ligands, termed ALKALs (i.e., ALK And LTK Ligands) has been identified (49, 50). Ligand binding triggers dimerization of ALK leading to trans-autophosphorylation of its intracellular domain and consequent activation. Activated ALK becomes a scaffold of sorts for a host of intracellular signaling effectors, leading to enactment of the PI3K-Akt, JAK3/STAT3, and Ras-ERK anti-apoptotic, pro-survival pathways (51, 52).

### 6.2 Putative role of ALK in chemoresistance of neuroblastoma

ALK was first discovered in a cell line from a patient with anaplastic large cell lymphoma. This cell line had a translocation in the genes for nucleophosmin (NPM) and ALK resulting in an NPM-ALK fusion protein (53). Since that discovery, not only have many different ALK fusion proteins been discovered in many different cancer types, but 9-10.5% of primary neuroblastomas have been found to carry mutations in the *ALK* gene, some of which are inherited and others, sporadic (53, 54). Of particular interest, the incidence of *ALK* mutations in recurrent neuroblastomas approaches 20% (55). These tumors tend to be particularly aggressive and particularly refractory to conventional chemotherapeutic agents. In fact, in patients with high-risk neuroblastoma, mutation and amplification of *ALK* are independent predictors of poor prognosis (55). In addition to the normal pro-survival, pro-migration activity of ALK, the mutant ALKs in recurrent neuroblastomas are thought to be pro-proliferative (56).

The discovery that gain-of-function mutations in *ALK* rendered neuroblastomas chemoresistant led to attempts to inhibit ALK function. The first ALK inhibitor approved for human use, crizotinib, was found to improve initial response of ALK mutant recurrent neuroblastomas but did not alter ultimate survival (53, 57). One explanation for this is drug-induced selection for neuroblastoma stem cells which secrete β-catenin, a protein known to bind to ALK and to thereby sterically inhibit the binding of crizotinib (56). In addition, the affinity of crizotinib for ALK varies depending on the relevant ALK mutation (53).

Several second generation ALK inhibitors have been approved and undergone clinical study for treatment of neuroblastoma. Pre-clinical studies of one of these, alectinib, proved promising (53) but it has not been tested in patients (58). Third generation ALK inhibitors include lorlatinib, which binds to a broad range of mutant ALKs, including those that do not bind crizotinib (59), and repotrectinib, which targets the active conformations of ALK, ROS1, and TRK (60). In an initial Phase I clinical study (59), lorlatinib demonstrated a 30% response rate in children with neuroblastoma. Its superior efficacy to crizotinib is thought to be related to its more potent cell cycle inhibition (61). Further clinical studies of lorlatinib are ongoing (57, 62). A preclinical study of using CAR T cells to target lorlatinib to ALK on neuroblastoma cells pointed out that another impediment to the success of such an approach and perhaps to ALK inhibitor use alone is the relatively low density of ALK molecules on the cell surface and the fact that, in the absence of ligand or inhibitor engagement, most of the ALK content of the cell is intracellular (63).

### 6.3 Overcoming the challenges of ALK-related chemoresistance in neuroblastoma

Inhibitors of anaplastic lymphoma kinase (ALK) are being studied in several ongoing Phase 1–3 clinical trials, particularly in patients with ALK mutation-driven solid tumors, including neuroblastoma (62). These largely involve optimizing dose, dosage schedule, therapeutic index, and matching of tumors and patients to specific drugs of promising existing ALK inhibitors.

But ALK-related chemoresistance in neuroblastoma involves tumors leveraging developmentally important molecules and processes to evade ALK inhibition. These evasive mechanisms sometimes create unique vulnerabilities that could be leveraged to therapeutic advantage (64).

One study by Berlak et al. (64) used genomic analysis of ALK inhibitor-resistant neuroblastomas to identify genomic changes associated with resistance to ALK inhibitors. They found that neuroblastoma cell lines rendered resistant by the selection pressure of chronic ALK inhibitor exposure had developed NF1 loss that resulted in upregulated signaling through the RAS-MAPK pathway and downregulated ERK-RAF feedback. These cells consequently became hypersensitive to treatment with MEK inhibitors. The authors conclude that such studies could suggest novel therapies that take advantage of the collateral consequences of the mechanisms neuroblastomas use to evade ALK inhibition. A 2026 preprint also demonstrates this principle with the use of an inhibitor of the RAS-MAPK pathway in RAS-mutant neuroblastoma (65). In an earlier study, Bruton’s tyrosine kinase (BTK) was shown to be upregulated and activated by aberrant ALK activation. Adjunctive inhibition of ALK and BTK synergistically enhances inhibition of neuroblastoma growth relative to the “sum” of inhibition of ALK or BTK alone in nude mouse-human xenograft model of neuroblastoma (66).

In addition, rather than the third-generation approach to ALK inhibition, which attempts to inhibit ALK across all mutation types and expression levels, one could create an ALK inhibitor or repressor library by making mutation-tailored decoy ligands or antisense or siRNAs, respectively, for each of the common oncogenic ALK mutations. This might be particularly effective in sporadic neuroblastoma-genic ALK mutations, as the mutated protein would only be expressed in the cancer cells. Choice of an individualized therapy for each patient’s tumor might be possible using this strategy.

Bergaggio and Chiarle (63) point out that, while CAR T cell therapy shows promise and affords antigen targeting in neuroblastoma, the challenge of targeting mutant ALK is its low density on the surface of the neuroblastoma cells. They suggest that adjunctive therapy, first with a high-affinity ALK inhibitor that would induce ALK dimerization and clustering on the cell surface without triggering ALK signaling, and subsequently with CAR T cells that target the relevant specific mutated ALK, now in high density on the cell surface, might be an effective approach to ALK-mediated chemoresistance in neuroblastoma.

Another mechanism of resistance of neuroblastoma cells to ALK inhibitors is their transition from neuroendocrine or adrenergic phenotype to mesenchymal or Schwann cell-like phenotype (67). The developmentally relevant plasticity and reversibility of cell phenotype underlies the potential for ALK inhibitors to select for and perhaps induce conversion of neuroendocrine neuroblastoma cells to mesenchymal neuroblastoma cells. Mesenchymal neuroblastoma cells do not express ALK and are therefore not sensitive to ALK inhibitors (67). It is conceivable that induction of neuroendocrine phenotype by pretreatment with steroids or differentiative retinoids could enhance the sensitivity of neuroblastomas to ALK inhibition.

Finally, the notion of neuroblastoma-selective introduction of loss-of-function mutations or editing of mutant *ALK* to render it wildtype in sequence using CRISPR-Cas9 technology is an intriguing idea for the future. The idea would be to introduce such an approach shortly after completion of therapy for the primary disease in order to “correct” ALK in minimal residual disease stem cells destined to constitute the recurrent neuroblastoma.

## 7 Developmentally relevant mediators of therapeutic resistance in neuroblastoma: histone deacetylase and enhancer of zeste homologue 2 histone modifiers

### 7.1 Developmental activation and function of HDAC and EZH2

Regulation and orchestration of transcription are critical functions during development. Ensuring proper timing and quantity of output is especially important for neural crest cells during migration, as the “match” between the changing milieu and the cellular protein complement means the difference between success and failure for these cells. The HDAC families of enzymes and the methyltransferase EZH2 play central roles in this process.

There are 4 classes of HDACs into which all of the 18 known HDACs are divided. The classes are defined largely by evolutionarily conserved structural characteristics. Class III, the sirtuins, is evolutionarily and structurally distinct from the other 3 classes and beyond the scope of this review.

Class I HDACs have been extensively studied primarily in the central nervous system and in craniofacial neural crest cell derivatives. They are involved in gating transcription during the epithelial-to-mesenchymal transition of central and cranial neural crest cells. In the peripheral nervous system, they regulate transcription in a way that facilitates pain signaling through the dorsal root ganglion when nerve injury occurs. They have also been hypothesized to be responsible for the transcription of a set of 118 genes that are dysregulated in Hirschsprung’s disease, a congenital intestinal disorder of migration of the cells of the myenteric plexus and submucosal plexus derived from the neural crest (68).

Class II HDACs are perhaps less well studied and understood. Class IIa HDACs have phosphorylation sites in their N-terminal domain and phosphorylation results in their trafficking from the nucleus to the cytoplasm of the cell. This adds to the complexity and plasticity of their function. They are thought to play roles in the brain in relation to sleep, hypersensitization to pain, and opioid addiction (68) and bind to the transcription factor, myocyte enhancer factor 2 (MEF2). They have not been implicated in the peripheral neural crest cell derivatives. Class IIb HDACs are primarily cytoplasmic, but they appear to play a role in modulation of chromatin architecture in muscle through their association with MEF2 and in some cancers. For example, in hepatocellular carcinoma, the histone methyltransferase, EZH2, represses expression of CXCL10 in an HDAC10-dependent manner (68).

HDAC11 is the only member of the Class IV HDACs. It is characterized by its zinc dependence and is primarily implicated in adipocyte function. It has not been specifically implicated in neural crest development (68).

EZH2 is an important histone methyltransferase known to influence development of the craniofacial and enteric neural crest derivatives. It represses gene expression as part of a polycomb repression complex with Suz12 and EED. It has been implicated in development of many neural crest derivatives, including melanocytes, astrocytes, and central and peripheral neurons. One pathway by which EZH2 influences neural crest-derived enteric submucosal plexus development has recently been discerned. The ciliary protein Kif7 downregulates Gli2. This downregulation enables sustained expression of the micro RNA miR124 which, in turn, downregulates the *Ezh2* gene. This facilitates neurogenesis in the intestine, as EZH2 and its polycomb repressor complex ordinarily downregulate genes involved in neural differentiation, while EZH2 acts as a transcriptional activator to upregulate genes involved in glial differentiation (69). Through histone H3K27me3 marks, EZH2 has been shown to repress expression of *Zic1*, *Pax3*, *Sox10, Ascl1, Phox2a, and Phox2b*, genes, genes that must be appropriately regulated to enable the normal balance of neural and glial differentiation of neural crest derivatives in the enteric nervous system (69, 70).

### 7.2 Putative role of HDAC and EZH2 in chemoresistance of neuroblastoma

The metabolic plasticity of neural crest cells is mirrored in the ability of neuroblastoma cells to metabolically reprogram themselves in the face of HDAC treatment. While this is not HDAC-induced generalized chemoresistance, it illustrates the power of cellular plasticity in evasion from chemotherapeutic attack. Cancer cells, with their high metabolic needs, often leverage the Warburg effect, the use of glycolysis through lactic acid, rather than through the tricarboxylic acid (TCA) cycle, to get ATP quickly, even if it is less efficient to do so. This is especially so of the most aggressive cancer cells and, in neuroblastoma particularly, this is the *MYCN*-overexpressing tumors. Treatment with HDAC inhibitors downregulates *MYCN* and the two rate-limiting enzymes at the beginning of the glycolytic pathway, hexokinase and glucose-6-phosphate isomerase. The cells rapidly adapt by shifting what glycolysis remains through the TCA cycle and upregulate enzymes involved in fatty acid oxidation. They thereby maintain their prodigious level of ATP production despite a relative block in anaerobic glycolysis and evade the effects of HDAC inhibition (71).

Deacetylation by HDAC and methylation by enzymes like EZH2 also render chromatin closed at specific sites and thereby have been shown to downregulate transcription of apoptosis-related genes and their pathways. Chang et al. (72) suggest that epigenetic modification of chromatin is responsible for shutting down apoptosis mediated through ATF4 and TRAIL, enhancing Akt- and ERK-mediated survival in pediatric tumors, including neuroblastoma.

*EZH2* is thought to be a target of the transcription factor N-myc (73) and to support oncogenicity and chemoresistance upon neural crest cells through methylation of H3K27 of putative tumor suppressor genes and effect on gene expression affecting the β-catenin, Ras, and nuclear factor kappa B pathways. Furthermore, EZH2 activates Src, a protein kinase upstream from focal adhesion kinase (FAK), a nonreceptor protein tyrosine kinase that is implicated in neuroblastoma tumorigenesis (74).

### 7.3 Overcoming the challenges of HDAC- and EZH2-related chemoresistance in neuroblastoma

In regard to the metabolic bypass mechanism used by neuroblastoma cells to escape from the effects of HDAC inhibition, screening of a National Cancer Institute panel of compounds demonstrated that the mTORC1 inhibitor sirolimus reversed the cells’ metabolic reprogramming and produced synergistic cell death *in vitro* and tumor shrinkage in a murine human xenograft model (71). This approach using vorinostat and temsirolimus has recently been studied in a small, Phase I clinical trial for diffuse pontine glioma (75).

In another adjunctive approach, Chang et al. (72) have demonstrated synergistic efficacy in neuroblastoma cell lines using an HDAC inhibitor, which facilitates production of ATF4 and TRAIL leading to caspase 8 activation, adjunctively with an imipradone, which inhibits signaling through Akt and ERK and decreases the mitochondrial protein ClpX, normally a protective force for mitochondrial health.

Other adjunctive approaches with HDAC inhibitors and dual-mechanistic approaches have been presented in preclinical studies (76, 77). In addition, HDAC8-specific inhibitors (members of Class I) appear to be particularly potent in preclinical studies of neuroblastoma (78).

In preclinical studies, inhibition of EZH2 results in decreased viability, motility, markers of stemness, and *in vivo* xenograft tumor growth in neuroblastoma (75).

An inhibitor of EZH2 began a Phase 1 trial for advanced solid tumors including neuroblastoma but was withdrawn from study because of safety concerns noted during ongoing Phase 2 trials in adults with solid tumors prompted by the development of secondary blood cancers in some study subjects (79, 80).

## 8 Discussion

Neuroblastoma is a cancer of cells derived from the developing neural crest cells destined to comprise the sympathetic nervous system. It is overwhelmingly a cancer of childhood, with an incidence of 1 in 7,000-10,000 live births. It accounts for 8% of childhood cancers and 15% of cancer mortality in children. Children with low-risk disease have a 5-year event-free survival rate of over 90% and children with intermediate-risk disease have a 5-year event-free survival rate of 85%. But for children with high-risk disease, the 5-year event-free survival rate is only 50% despite multimodal therapy (81).

The treatment-refractory nature of disseminated neuroblastoma is, in part, related to the intratumoral and inter-metastatic site heterogeneity and developmentally-mediated plasticity of molecular, proteomic, and cellular phenotypes of neuroblastoma cells and their environment. Even within a single patient’s primary tumor there are neighborhoods of cells of differing stages of differentiation with different transcriptomic expression profiles and correspondingly different morphologies and cellular behavior profiles. These cells range from adrenergic, neuroendocrine cells to Schwann-like, stromal cells, with intermediate, stem-like cells between them. They exhibit facility in bidirectional transition from one phenotype to another and, with it, the ability to shift metabolic profiles to evade enzymic inhibition and substrate deprivation. This heterogeneity gives neuroblastomas a developmentally-grounded ability to evade targeted chemotherapeutic attack by evolving *in situ* different propensities for and resistance to proliferation, migration, and apoptosis enactment (5, 7, 32, 33, 71).

Adding to the chemotherapeutic resistance afforded neuroblastoma by cellular heterogeneity is the complexity of neurotrophin receptor cognate ligands, cooperative binding to p75NTR, and dependence of signaling pathway effectors on the cell membrane and extracellular environment in which the receptors sit. For example, in general, high TrkA expression bodes well while high TrkB expression bodes poorly for prognosis of neuroblastoma patients. However, when p75NTR binds to TrkB, the affinity of TrkB for mature BDNF increases, encouraging angiogenesis in and metastasis of tumor cells. But when p75NTR stoichiometrically overtakes TrkB, the relevant ligand becomes proBDNF and apoptosis, rather than angiogenesis and metastasis, ensues (7).

Transcription factors constitute a sizable fraction of the contributors to oncogenic transformation of neural crest cells. They are challenging as therapeutic targets because of the diversity of their downstream activities that make off-target effects likely; their lack of an ordered, function-relatable structure; the fact that, unlike enzymes, they do not have a readily identifiable substrate binding site or trigger an inhibitable reaction; and the presence and required involvement of multiple cofactors in their function. However, recently, screening of a chemical library revealed one compound that decreased the cellular half-life of N-myc, a transcription factor and oncoprotein associated with aggressive neuroblastoma. Preclinical laboratory studies of this compound suggest that it decreases growth of tumors in a human neuroblastoma murine xenograft model (82). In addition, attempts to leverage therapeutically inhibition of methylation and consequent accumulation of the oncoprotein N-myc and the transcription cofactor EYA1 have shown some promise in murine and *in vitro* human neuroblastoma (22, 23, 47). Inhibition of PRMTs, the enzymes responsible for protein arginine methylation, appeared efficacious and non-toxic in animal models but off-target effects have limited dose escalation to the point of efficacy and have proved problematic in adult cancer patients. One approach to this challenge would be to employ antisense RNA or siRNA constructs to downregulate the relevant PRMT(s) in a particular tumor. Human neuroblastoma cell line studies most heavily implicate PRMT1 and, somewhat less heavily, PRMT5 in this mechanism (22, 23). Antisense and siRNA constructs would provide highly selective knockdown or knockout of these enzymes and perhaps could be derivatized to make them specifically target the sympathoblastic neuroblastoma cells of the tumors.

With regard to oncogenic histone modification, an inhibitor of epigenetic histone methylation, EZH2, has proved to be intolerably toxic and so has been withdrawn from investigation (80). Inhibitors of HDAC, which prevent deacetylation of histones, are currently the subject of Phase 1 clinical trials in children with neuroblastoma and other solid tumors (83). In addition, several adjunctive approaches to routing cells through apoptosis-inducing or HDAC-dependent signaling pathways or blocking metabolic bypass strategies for resistance to HDAC inhibition, and then inhibiting HDAC activity have been proposed (68, 84, 85). These preclinical approaches illustrate the power of steering tumor cells in the direction of vulnerability and susceptibility to a particular chemotherapeutic agent and mechanism. The use of newer molecular methods for doing so durably and at the proximal source of the phenotype is an emerging and promising strategy. For example, interference with miR124 production or activity may interfere with the EZH2 pathway downstream of direct inhibition and may avoid some of the toxic effects of direct EZH2 inhibition.

For the minority of neuroblastoma patients with familial mutations of ALK, inhibitors of this kinase appear quite promising and are under clinical study (62). In the future, proactive identification of those at risk might afford use of *in vivo* CRISPR-Cas9 technology to edit the gain-of-function mutation that makes this tyrosine kinase oncogenic and to thereby prevent or arrest in early stages neuroblastoma. In addition, in situations in which neuroblastomas evade ALK inhibitor therapy by adoption of a mesenchymal phenotype, pretreatments that force conversion of neuroblastoma cells to a neuroendocrine phenotype (i.e., mesenchymal-to-epithelial transition) would maximize ALK expression and could then be followed by ALK inhibitors to greater therapeutic effect than otherwise would be the case.

### 8.1 Strengths and limitations of this review

This presentation of the challenges of designing effective therapies for neuroblastoma that leverage its expression and signaling activity of neurotrophin receptors, transcription factor targets, tyrosine receptor kinases, and histone modifying enzymes does not represent a systematic review of all the proposed therapeutic approaches to neuroblastoma. It is possible, therefore, exploitation or inhibition of other developmentally relevant mechanisms could contribute to or prove fruitful in overcoming chemotherapeutic resistance in clinical therapy for this tumor. However, the related strength of this strategy is that this review focuses in some depth on examples that illustrate the overarching concept developmental plasticity and consequent cellular and environmental heterogeneity contribute to the chemoresistance of neuroblastoma, and likely other developmental malignancies. The studies and therapeutic approaches presented in this review exemplify the relationships between normal and oncogenic development in the neural crest and to incite discussion and innovation towards the next generation of therapeutic approaches to neuroblastoma, an all-too-commonly lethal cancer of childhood.

## 9 Conclusion

Neuroblastoma is the single most common cancer of childhood. It arises from the cells of the neural crest as they migrate out to become the sympathetic nervous system (4). The tumor expression profile of neuroblastomas illustrates both the developmental plasticity of the neural crest cells as they navigate through widely differing environments during their epithelial-to-mesenchymal transition and the power of that plasticity in conferring malignant and chemoresistant character upon these cells. Attempts to overcome chemoresistance in neuroblastomas have included chemotherapeutic agents and adjuncts aimed at neurotrophic signaling, gain-of-function mutants of other tyrosine kinases, developmentally relevant transcription factors and their targets, and histone modifying enzymes that contribute to oncogenicity and chemoresistance.

Several features of neuroblastoma have made these therapeutic approaches challenging (7, 33). The engagement of the same effectors and mechanisms in normal development as in chemoresistance makes achieving safety of potential targeted therapies challenging. The plasticity of developing sympathoblast expression profiles, physiology, and morphological lineage allows for evasion of chemotherapeutic attack aimed at developmentally relevant targets in neuroblastoma cells. Future efforts will likely take advantage of newer molecular technologies and their potential to be adapted to *in vivo* human use and continue to involve both individualization of therapy and a rotating (to attack based on selection for chemo-evasive spontaneous changes in cellular characteristics) and adjunctive (to steer changes in cellular characteristics in the direction of chemo-vulnerability) therapeutic cocktail approach to the intratumoral evolution of developmental and oncogenic signaling characteristic of neuroblastoma.

## References

[1] Axelson H Fredlund E Ovenberger M Landberg G Påhlman S . Hypoxia-induced dedifferentiation of tumor cells--a mechanism behind heterogeneity and aggressiveness of solid tumors. Semin Cell Dev Biol. (2005) 16:554–63. doi: 10.1016/j.semcdb.2005.03.007 16144692

[2] Knudson AG . Cancer genetics. Am J Med Genet. (2002) 111:96–102. doi: 10.1002/ajmg.10320 12124744

[3] Ritter KE Durbin AD . Lineage-selective dependencies in pediatric cancers. Cold Spring Harb Perspect Med. (2025) 15:a041573. doi: 10.1101/cshperspect.a041573 38806246 PMC11882016

[4] De Bona A Barbieri M Rinaldi N Esposito S . Neuroblastoma in childhood: biological insights, risk stratification, and advances in multimodal therapy. J Clin Med. (2026) 15:1101. doi: 10.3390/jcm15031101 41682783 PMC12898582

[5] Tang W Bronner ME . Neural crest lineage analysis: from past to future trajectory. Development. (2020) 147:dev193193. doi: 10.1242/dev.193193 33097550 PMC7595686

[6] Ponzoni M Bachetti T Corrias MV Brignole C Pastorino F Calarco E . Recent advances in the developmental origin of neuroblastoma: an overview. J Exp Clin Cancer Res. (2022) 41:92. doi: 10.1186/s13046-022-02281-w 35277192 PMC8915499

[7] Siddi C Balla J Fadda P Dedoni S . Epigenetic regulation of Trk receptors and neurotrophic signalling in neuroblastoma: Mechanisms, plasticity, and therapeutic opportunities. Int J Mol Sci. (2026) 27:3238. doi: 10.3390/ijms27073238 41977421 PMC13073776

[8] Cortazzo MH Kassis ES Sproul KA Schor NF . Nerve growth factor (NGF)-mediated protection of neural crest cells from antimitotic agent-induced apoptosis: the role of the low-affinity NGF receptor. J Neurosci. (1996) 16:3895–9. doi: 10.1523/JNEUROSCI.16-12-03895.1996 8656283 PMC6578614

[9] Hickman FE Stanley EM Carter BD . Neurotrophin responsiveness of sympathetic neurons is regulated by rapid mobilization of the p75 receptor to the cell surface through TrkA activation of Arf6. J Neurosci. (2018) 38:5606–19. doi: 10.1523/JNEUROSCI.0788-16.2018 29789375 PMC6001035

[10] Yan C Liang Y Nylander KD Schor NF . TrkA as a life and death receptor: receptor dose as a mediator of function. Cancer Res. (2002) 62:4867–75. 12208732

[11] Martin-Zanca D Barbacid M Parada LF . Expression of the trk proto-oncogene is restricted to the sensory cranial and spinal ganglia of neural crest origin in mouse development. Genes Dev. (1990) 4:683–94. doi: 10.1101/gad.4.5.683 2379826

[12] Niewiadomska G Mietelska-Porowska A Mazurkiewicz M . The cholinergic system, nerve growth factor and the cytoskeleton. Behav Brain Res. (2011) 221:515–26. doi: 10.1016/j.bbr.2010.02.024 20170684

[13] Rifkin JT Todd VJ Anderson LW Lefcort F . Dynamic expression of neurotrophin receptors during sensory neuron genesis and differentiation. Dev Biol. (2000) 227:465–80. doi: 10.1006/dbio.2000.9841 11071767

[14] Kasemeier-Kulesa JC Spengler JA Muolo CE Morrison JA Woolley TE Schnell S . The embryonic trunk neural crest microenvironment regulates the plasticity and invasion of human neuroblastoma via TrkB signaling. Dev Biol. (2021) 480:78–90. doi: 10.1016/j.ydbio.2021.08.007 34416224

[15] Straub JA Sholler GL Nishi R . Embryonic sympathoblasts transiently express TrkB *in vivo* and proliferate in response to brain-derived neurotrophic factor *in vitro*. BMC Dev Biol. (2007) 7:10. doi: 10.1186/1471-213X-7-10 17309801 PMC1805739

[16] Zahavi EE Steinberg N Altman T Chein M Joshi Y Gradus-Pery T . The receptor tyrosine kinase TrkB signals without dimerization at the plasma membrane. Sci Signal. (2018) 11:eaao4006. doi: 10.1126/scisignal.aao4006 29739881

[17] Wislet S Vandervelden G Rogister B . From neural crest development to cancer and vice versa: how p75^ntr^ and (pro)neurotrophins could act on cell migration and invasion? Front Mol Neurosci. (2018) 11:244. doi: 10.3389/fnmol.2018.00244 30190671 PMC6115613

[18] Smedfors G Olson L Karlsson TE . A Nogo-like signaling perspective from birth to adulthood and in old age: brain expression patterns of ligands, receptors and modulators. Front Mol Neurosci. (2018) 11:42. doi: 10.3389/fnmol.2018.00042 29520216 PMC5827527

[19] Rogers DA Schor NF . Kidins220/ARMS is expressed in neuroblastoma tumors and stabilizes neurotrophic signaling in a human neuroblastoma cell line. Pediatr Res. (2013) 74:517–24. doi: 10.1038/pr.2013.146 23999075 PMC3968798

[20] Rogers DA Schor NF . Kidins220/ARMS depletion is associated with the neural-to Schwann-like transition in a human neuroblastoma cell line model. Exp Cell Res. (2013) 319:660–9. doi: 10.1016/j.yexcr.2012.12.027 23333500

[21] Bassili M Birman E Schor NF Saragovi HU . Differential roles of Trk and p75 neurotrophin receptors in tumorigenesis and chemoresistance *ex vivo* and *in vivo*. Cancer Chemother Pharmacol. (2010) 65:1047–56. doi: 10.1007/s00280-009-1110-x 19701634 PMC4315186

[22] Li X Eberhardt A Hansen JN Bohmann D Li H Schor NF . Methylation of the phosphatase-transcription activator EYA1 by protein arginine methyltransferase 1: Mechanistic, functional, and structural studies. FASEB J. (2017) 31:2327–39. doi: 10.1096/fj.201601050RR 28213359

[23] Eberhardt A Hansen JN Koster J Lotta LT Wang S Livingstone E . Protein arginine methyltransferase 1 is a novel regulator of MYCN in neuroblastoma. Oncotarget. (2016) 7:63629–39. doi: 10.18632/oncotarget.11556 27571165 PMC5325390

[24] Cappabianca L Ruggieri M Sebastiano M Sbaffone M Martelli I Ruggeri P . Molecular characterization and inhibition of a novel stress-induced mitochondrial protecting role for misfolded TrkAIII in human SH-SY5Y neuroblastoma cells. Int J Mol Sci. (2024) 25:5475. doi: 10.3390/ijms25105475 38791513 PMC11122047

[25] Farina AR Cappabianca L Ruggeri P Gneo L Pellegrini C Fargnoli MC . The oncogenic neurotrophin receptor tropomyosin-related kinase variant, TrkAIII. J Exp Clin Cancer Res. (2018) 37:119. doi: 10.1186/s13046-018-0786-3 29914559 PMC6006588

[26] Wang Y Zhao Z Wang J Song S Zhao C Qv C . Advances in multi-omics research on neuroblastoma. Front Pediatr. (2026) 14:1739376. doi: 10.3389/fped.2026.1739376 42078544 PMC13128637

[27] Tang XX Evans AE Zhao H Cnaan A Brodeur GM Ikegaki N . Association among EPHB2, TrkA, and MYCN expression in low-stage neuroblastomas. Med Pediatr Oncol. (2001) 36:80–2. doi: 10.1002/1096-911X(20010101)36:1<80::AID-MPO1019>3.0.CO;2-N 11464911

[28] Xiong X Li Y Liu L Qi K Zhang C Chen Y . Arsenic trioxide induces cell cycle arrest and affects Trk receptor expression in human neuroblastoma SK-N-SH cells. Biol Res. (2018) 51:18. doi: 10.1186/s40659-018-0167-6 29898774 PMC5998579

[29] Wu Y Zhang J . Study on differentially expressed genes between Stage M and Stage MS neuroblastoma. Front Oncol. (2023) 12:1083570. doi: 10.3389/fonc.2022.1083570 36713522 PMC9880530

[30] Hansen JN Lotta LT Eberhardt A Schor NF Li X . EYA1 expression and subcellular localization in neuroblastoma and its association with prognostic markers. J Cancer Res Ther (Manch). (2016) 4:11–8. doi: 10.14312/2052-4994.2016-3 28713571 PMC5507068

[31] Cappabianca L Sebastiano M Ruggieri M Sbaffone M Zelli V Farina AR . Doxorubicin-induced trkAIII activation: A selection mechanism for resistant dormant neuroblastoma cells. Int J Mol Sci. (2022) 23:10895. doi: 10.3390/ijms231810895 36142807 PMC9503591

[32] Kyriakoudi SA Chatzi D Dermitzakis I Gargani S Manthou ME Meditskou S . Genetic identity of neural crest cell differentiation in tissue and organ development. Front Biosci (Landmark Ed). (2024) 29:261. doi: 10.31083/j.fbl2907261 39082344

[33] Stundl J Desingu Rajan AR Bronner ME . Neural crest gene regulatory networks as drivers of development, diversification and disease. Nat Rev Mol Cell Biol. (2026) 27:467–85. doi: 10.1038/s41580-026-00949-1 41781486 PMC13085950

[34] Westermark UK Wilhelm M Frenzel A Henriksson MA . The MYCN oncogene and differentiation in neuroblastoma. Semin Cancer Biol. (2011) 21:256–66. doi: 10.1016/j.semcancer.2011.08.001 21849159

[35] Makimoto A Fujisaki H Matsumoto K Takahashi Y Cho Y Morikawa Y . Retinoid therapy for neuroblastoma: Historical overview, regulatory challenges, and prospects. Cancers. (2024) 16:544. doi: 10.3390/cancers16030544 38339295 PMC10854948

[36] Yang YP Wang S Li X Schor NF . Cell line-dependent variability of coordinate expression of p75NTR and CRABP1 and modulation of effects of fenretinide on neuroblastoma cells. Oxid Med Cell Longev. (2016) 2016:7568287. doi: 10.1155/2016/7568287 26843908 PMC4710897

[37] Panosyan WS Panosyan DE . Immunoliposomes for neuroblastoma: Review of the past experience and design of a novel nanoparticle. Anticancer Res. (2024) 44:4665–75. doi: 10.21873/anticanres.17294 39477321

[38] Baxi A Jourdeuil K Cox TC Clouthier DE Tavares ALP . Transcriptomic analysis reveals the role of SIX1 in mouse cranial neural crest patterning and bone development. Dev Dyn. (2023) 252:1303–15. doi: 10.1002/dvdy.597 37183792 PMC10592572

[39] Wu Z Rao Y Zhang S Kim EJ Oki S Harada H . Cis-control of Six1 expression in neural crest cells during craniofacial development. Dev Dyn. (2019) 248:1264–72. doi: 10.1002/dvdy.109 31464047

[40] Xu PX Zheng W Huang L Maire P Laclef C Silvius D . Six1 is required for the early organogenesis of mammalian kidney. Development. (2003) 130:3085–94. doi: 10.1242/dev.00536 12783782 PMC3872112

[41] Sears TK Angelastro JM . The transcription factor ATF5: role in cellular differentiation, stress responses, and cancer. Oncotarget. (2017) 8:84595–609. doi: 10.18632/oncotarget.21102 29137451 PMC5663623

[42] Gu L Yang T Zhang J Wu J Fan Q Zhang Y . The TBX18/SIX1 Transcriptional circuit maintains stemness and EMT states to promote radioresistance in ESCC. Cancers (Basel). (2026) 18:2700. doi: 10.3390/cancers18162700 42650011 PMC13510439

[43] Liao Y Yin S Peng EY Wu J Yao Y He W . The GCN5L2-SIX1 complex drives glycolysis to promote progression of triple-negative breast cancer. Cancer Lett. (2026) 659:218770. doi: 10.1016/j.canlet.2026.218770 42601012

[44] Zhu G Piao Z Gao Y Cong X Wei P Han A . Glycolytic reprogramming and the EMT drive cervical cancer progression via the NQO1/HIF1α-SIX1 axis. iScience. (2026) 29:117007. doi: 10.1016/j.isci.2026.117007 42571403 PMC13452244

[45] Tong G Qi H . GRP78 Drives NSCLC stemness and EMT via a SIX1/β-Catenin signaling axis. Drug Dev Res. (2026) 87:e70359. doi: 10.1002/ddr.70359 42546137 PMC13432796

[46] Banerjee D Boboila S Okochi S Angelastro JM Kadenhe-Chiweshe AV Lopez G . Activating Transcription Factor 5 promotes neuroblastoma metastasis by inducing anoikis resistance. Cancer Res Commun. (2023) 3:2518–30. doi: 10.1158/2767-9764.CRC-23-0154 38014922 PMC10714915

[47] Hua ZY Hansen JN He M Dai SK Choi Y Fulton MD . PRMT1 promotes neuroblastoma cell survival through ATF5. Oncogenesis. (2020) 9:50. doi: 10.1038/s41389-020-0237-9 32415090 PMC7229216

[48] El-Khoueiry AB Clarke J Neff T Crossman T Ratia N Rathi C . Phase 1 study of GSK3368715, a type I PRMT inhibitor, in patients with advanced solid tumors. Br J Cancer. (2023) 129:309–17. doi: 10.1038/s41416-023-02276-0 37237172 PMC10338470

[49] Fadeev A Mendoza-Garcia P Irion U Guan J Pfeifer K Wiessner S . ALKALs are *in vivo* ligands for ALK family receptor tyrosine kinases in the neural crest and derived cells. Proc Natl Acad Sci USA. (2018) 115:E630–8. doi: 10.1073/pnas.1719137115 29317532 PMC5789956

[50] Vieceli FM Bronner ME . Leukocyte receptor tyrosine kinase interacts with secreted midkine to promote survival of migrating neural crest cells. Development. (2018) 145:dev164046. doi: 10.1242/dev.164046 30228102 PMC6215395

[51] Moreno MM Barrell WB Godwin A Guille M Liu KJ . Anaplastic lymphoma kinase (alk), a neuroblastoma associated gene, is expressed in neural crest domains during embryonic development of Xenopus. Gene Expr Patterns. (2021) 40:119183. doi: 10.1016/j.gep.2021.119183 34020009 PMC7616747

[52] Aboualizadeh F Yao Z Guan J Drecun L Pathmanathan S Snider J . Mapping the phospho-dependent ALK interactome to identify novel components in ALK signaling. J Mol Biol. (2021) 433:167283. doi: 10.1016/j.jmb.2021.167283 34606829

[53] Alam MW Borenäs M Lind DE Cervantes-Madrid D Umapathy G Palmer RH . Alectinib, an anaplastic lymphoma kinase inhibitor, abolishes ALK activity and growth in ALK-positive neuroblastoma cells. Front Oncol. (2019) 9:579. doi: 10.3389/fonc.2019.00579 31334113 PMC6625372

[54] Allinson LM Potts A Goodman A Bown N Bashton M Thompson D . Loss of ALK hotspot mutations in relapsed neuroblastoma. Genes Chromosomes Cancer. (2022) 61:747–53. doi: 10.1002/gcc.23093 36029175 PMC9826054

[55] Bellini A Pötschger U Bernard V Lapouble E Baulande S Ambros PF . Frequency and prognostic impact of *ALK* amplifications and mutations in the European Neuroblastoma Study Group (SIOPEN) high-risk neuroblastoma trial (HR-NBL1). J Clin Oncol. (2021) 39:3377–90. doi: 10.1200/JCO.21.00086 34115544 PMC8791815

[56] Alshareef A Gupta N Zhang HF Wu C Haque M Lai R . High expression of β-catenin contributes to the crizotinib resistant phenotype in the stem-like cell population in neuroblastoma. Sci Rep. (2017) 7:16863. doi: 10.1038/s41598-017-17319-9 29203817 PMC5715105

[57] Brenner AK Gunnes MW . Therapeutic targeting of the anaplastic lymphoma kinase (ALK) in neuroblastoma-a comprehensive update. Pharmaceutics. (2021) 13:1427. doi: 10.3390/pharmaceutics13091427 34575503 PMC8470592

[58] Bheemavarapu B Khalil M Rehman A Javid S Cyrus F Qayyum SN . Integration of ALK gene mutations and targeted therapies in pediatric high-risk neuroblastoma: advancements in precision oncology. Ann Med Surg (Lond). (2025) 87:6470–9. doi: 10.1097/MS9.0000000000003697 41181401 PMC12577807

[59] Goldsmith KC Park JR Kayser K Malvar J Chi YY Groshen SG . Lorlatinib with or without chemotherapy in ALK-driven refractory/relapsed neuroblastoma: phase 1 trial results. Nat Med. (2023) 29:1092–102. doi: 10.1038/s41591-023-02297-5 37012551 PMC10202811

[60] Cervantes-Madrid D Szydzik J Lind DE Borenäs M Bemark M Cui J . Repotrectinib (TPX-0005), effectively reduces growth of ALK driven neuroblastoma cells. Sci Rep. (2019) 9:19353. doi: 10.1038/s41598-019-55060-7 31852910 PMC6920469

[61] Matkar S East MP Stuhlmiller TJ Witek GM Farrel A Pastor S . Kinome reprogramming of G2/M kinases and repression of MYCN contribute to superior efficacy of lorlatinib in ALK-driven neuroblastoma. Mol Cancer Ther. (2025) 24:1389–401. doi: 10.1158/1535-7163.MCT-24-0684 39907037 PMC12322148

[62] National Library of Medicine . NCT02650401, NCT03107988, NCT00939770, NCT03126916, and NCT03155620. Available online at: https://clinicaltrials.gov/search?cond=neuroblastoma&intr=ALK%20inhibitor&viewType=Card (Accessed June 29, 2026).

[63] Bergaggio E Chiarle R . Impact of ALK inhibitors to potentiate ALK.CAR-T therapy in neuroblastoma. Clin Transl Med. (2024) 14:e1732. doi: 10.1002/ctm2.1732 38877641 PMC11178513

[64] Berlak M Tucker E Dorel M Winkler A McGearey A Rodriguez-Fos E . Mutations in ALK signaling pathways conferring resistance to ALK inhibitor treatment lead to collateral vulnerabilities in neuroblastoma cells. Mol Cancer. (2022) 21:126. doi: 10.1186/s12943-022-01583-z 35689207 PMC9185889

[65] Hill RD Dalton KM Kurupi R Slaughter JM Roberts J Xing Y . Daraxonrasib (RMC-6236) is an effective targeted therapy for *RAS* -mutant neuroblastoma. In: Biorxiv [Preprint] (2026). p. 2026.02.19.706849. doi: 10.64898/2026.02.19.706849

[66] Li T Deng Y Shi Y Tian R Chen Y Zou L . Bruton’s tyrosine kinase potentiates ALK signaling and serves as a potential therapeutic target of neuroblastoma. Oncogene. (2018) 37:6180–94. doi: 10.1038/s41388-018-0397-7 30013190

[67] Westerhout EM Hamdi M Stroeken P Nowakowska NE Lakeman A van Arkel J . Mesenchymal-type neuroblastoma cells escape ALK inhibitors. Cancer Res. (2022) 82:484–96. doi: 10.1158/0008-5472.CAN-21-1621 34853072

[68] Van Bree BA Eichner LJ . The impactful role of the HDACs in the regulation of gene expression and as targets for disease therapy. Sci Adv. (2026) 12:eaee7295. doi: 10.1126/sciadv.aee7295 42247509 PMC13267311

[69] Lai FP Li Z Zhou T Leung AOW Lau ST Lui KN . Ciliary protein Kif7 regulates Gli and Ezh2 for initiating the neuronal differentiation of enteric neural crest cells during development. Sci Adv. (2021) 7:eabf7472. doi: 10.1126/sciadv.abf7472 34644112 PMC8514102

[70] Kim H Langohr IM Faisal M McNulty M Thorn C Kim J . Ablation of Ezh2 in neural crest cells leads to aberrant enteric nervous system development in mice. PloS One. (2018) 13:e0203391. doi: 10.1371/journal.pone.0203391 30169530 PMC6118393

[71] Bishayee K Nazim UM Kumar V Kang J Kim J Huh SO . Reversing the HDAC-inhibitor mediated metabolic escape in MYCN-amplified neuroblastoma. BioMed Pharmacother. (2022) 150:113032. doi: 10.1016/j.biopha.2022.113032 35486977

[72] Chang WI Honeyman JN Zhang J Lin C Sharma A Zhou L . Novel combination of imipridones and histone deacetylase inhibitors demonstrate cytotoxic effect through integrated stress response in pediatric solid tumors. Am J Cancer Res. (2023) 13:6241–55. 38187038 PMC10767354

[73] Chen L Alexe G Dharia NV Ross L Iniguez AB Conway AS . CRISPR-Cas9 screen reveals a MYCN-amplified neuroblastoma dependency on EZH2. J Clin Invest. (2018) 128:446–62. doi: 10.1172/JCI90793 29202477 PMC5749506

[74] Bownes LV Williams AP Marayati R Stafman LL Markert H Quinn CH . EZH2 inhibition decreases neuroblastoma proliferation and *in vivo* tumor growth. PloS One. (2021) 16:e0246244. doi: 10.1371/journal.pone.0246244 33690617 PMC7942994

[75] Stitzlein LM Baig MU Chandra J McGovern S Paulino A Ketonen LM . Phase I study of vorinostat and temsirolimus in newly diagnosed or progressive diffuse intrinsic pontine glioma. Pediatr Blood Cancer. (2025) 72:e31619. doi: 10.1002/pbc.31619 40000388

[76] Cheung BB Kleynhans A Mittra R Kim PY Holien JK Nagy Z . A novel combination therapy targeting ubiquitin-specific protease 5 in MYCN-driven neuroblastoma. Oncogene. (2021) 40:2367–81. doi: 10.1038/s41388-021-01712-w 33658627 PMC8016666

[77] Kong Y Li S Xue X Liu H Fu R Gao Y . Design, synthesis and biological evaluation of ALK/HDAC dual-targeting agents. Bioorg Med Chem Lett. (2026) 133:130495. doi: 10.1016/j.bmcl.2025.130495 41389892

[78] Cho H Lee E Kim J Shin S Kim YJ Lee H . Discovery of organosulfur-based selective HDAC8 inhibitors with anti-neuroblastoma activity. Eur J Pharm Sci. (2024) 203:106921. doi: 10.1016/j.ejps.2024.106921 39357770

[79] National Cancer Institute . NCT03155620: Targeted Therapy Directed by Genetic Testing in Treating Pediatric Patients With Relapsed or Refractory Advanced Solid Tumors, non-Hodgkin Lymphomas, or Histiocytic Disorders (the Pediatric MATCH Screening Trial). Available online at: https://clinicaltrials.gov/study/NCT03155620?cond=neuroblastoma&viewType=Card&intr=EZH2%20inhibitor&rank=1 (Accessed June 29, 2026).

[80] Britt P . Tazemetostat withdrawn from all trials and markets due to safety concerns Targeted Oncol (2026). Available online at: https://www.targetedonc.com/view/tazemetostat-withdrawn-from-all-trials-and-markets-due-to-safety-concerns (Accessed June 29, 2026).

[81] Kotagal M Grant CN Brown EG Lal DR Newman EA . Neuroblastoma. Surg Oncol Clin N Am. (2026) 35:715–29. doi: 10.1016/j.soc.2026.04.012 42686228

[82] Miao Y Chen H Li Y Li L Ye J Zhang J . A small molecule selectively targets N-Myc to suppress neuroblastoma cancer progression. Int J Biol Sci. (2025) 21:4895–907. doi: 10.7150/ijbs.97195 40860201 PMC12374823

[83] National Institutes of Health Clinical Center . NCT00020579: MS-275 in Treating Patients With Advanced Solid Tumors or Lymphoma. Available online at: https://clinicaltrials.gov/study/NCT00020579?cond=neuroblastoma&viewType=Card&intr=HDAC%20inhibitor&srank=1 (Accessed June 29, 2026).

[84] Lundberg KI Treis D Johnsen JI . Neuroblastoma heterogeneity, plasticity, and emerging therapies. Curr Oncol Rep. (2022) 24:1053–62. doi: 10.1007/s11912-022-01270-8 35362827 PMC9249718

[85] Gautam N Wang S Üren A Chapagain PP Adhikari NP Tiwari PB . Mapping the interactions among class IIa histone deacetylases and myocyte enhancer factor 2s. J Chem Inf Model. (2025) 65:6249–60. doi: 10.1021/acs.jcim.5c00858 40478640 PMC12199305

